# Remarkable Effect of [Li(G4)]TFSI Solvate Ionic Liquid (SIL) on the Regio- and Stereoselective Ring Opening of α-Gluco Carbasugar 1,2-Epoxides

**DOI:** 10.3390/molecules24162946

**Published:** 2019-08-14

**Authors:** Sebastiano Di Pietro, Vittorio Bordoni, Andrea Mezzetta, Cinzia Chiappe, Giovanni Signore, Lorenzo Guazzelli, Valeria Di Bussolo

**Affiliations:** 1 Dipartimento di Farmacia, Università di Pisa, Via Bonanno 33, 56126 Pisa, Italy; 2 Present address: Max Planck Institute of Colloids and Interfaces, Am Mühlen- berg 1, 14476 Potsdam, Germany; 3 Fondazione Pisana per la Scienza, via F. Giovannini 13, San Giuliano Terme (PI), 56017 Pisa, Italy; 4 Dipartimento di Chimica e Chimica Industriale, Università di Pisa, Via G. Moruzzi 3, 56124 Pisa, Italy

**Keywords:** solvate ionic liquids (SILs), [Li(G3)]TFSI, [Li(G4)]TFSI, carbasugars, α-gluco 1,2-epoxide

## Abstract

Carba analogues of biologically relevant natural carbohydrates are promising structures for the development of future drugs endowed with enhanced hydrolytic stability. An open synthetic challenge in this field is the optimization of new methodologies for the stereo- and regioselective opening of α-gluco carbasugar 1,2-epoxides that allow for the preparation of pseudo mono- and disaccharides of great interest. Therefore, we investigated the effect of Lewis acids and solvate ionic liquids (SILs) on the epoxide ring opening of a model substrate. Of particular interest was the complete stereo- and regioselectivity, albeit limited to simple nucleophiles, toward the desired C(1) isomer that was observed using LiClO_4_. The results obtained with SILs were also remarkable. In particular, Li[NTf_2_]/tetraglyme (**[Li(G4)]TFSI**) was able to function as a Lewis acid and to direct the attack of the nucleophile preferentially at the pseudo anomeric position, even with a more complex and synthetically interesting nucleophile. The regioselectivity observed for LiClO_4_ and **[Li(G4)]TFSI** was tentatively ascribed to the formation of a bidentate chelating system, which changed the conformational equilibrium and ultimately permitted a trans-diaxial attack on C(1). To the best of our knowledge, we report here the first case in which SILs were successfully employed in a ring-opening process of epoxides.

## 1. Introduction

Ionic liquids (ILs) are salts with low melting temperatures that have gained significant research attention as pure compounds, in their mono- or dicationic forms [1,2,3], as well as mixtures [4,5] for a wide variety of applications spanning from materials science [6,7,8,9] to analytical chemistry [10,11] and biological science [12].

The favorable characteristics of ILs also make them extremely interesting for carrying out reactions in carbohydrate chemistry [13,14,15].

Recently, a range of novel solvate ionic liquids (SILs) have been identified [16,17,18,19,20,21,22,23,24]. SILs are a sub-class of ILs, consisting of a metal salt and a stoichiometric amount of a coordinating ligand that form stable one-to-one mixtures of complexed cations and counter ions [25]. The most widely investigated SILs are probably based on oligoethers (glymes) and metal salts. The equimolar mixtures of glymes and certain lithium salts are indeed liquids at room temperature and, similar to many ILs, are endowed with low volatility, high ionic conductivities and high electrochemical stability, thus justifying the proposed name (solvate ionic liquids).

The potential use of SILs as new electrolytes for lithium-based batteries [26,27,28] or as electrolytes for thermoelectrochemical cells [29] has been studied in depth, while only very few applications in organic transformations have been reported [30]. Recently, some significant examples have focused on the use of SILs in Diels Alder reactions [30], as organo-catalysts in aldol reactions with (S)-proline [31], in the [2 + 2] cycloaddition formation of dienes [30], and in the preparation of α-aminophosphonates [32]. Although ILs have been employed as media for the ring opening of epoxides [33,34,35,36], no data are available for SILs.

**Scheme 1:**
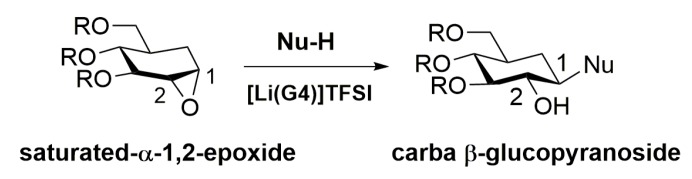
Regio- and stereoselective addition of a generic nucleophile in **[Li(G4)]TFSI** to a carbapyranose-α-1,2-epoxide.

Herein, we report an unexplored attractive application of these SILs as solvents/Lewis acids that are able to efficiently promote the ring opening of epoxides due to the coordination between the substrate and the cationic species present into the glyme [37]. In particular, in the context of a research program concerning the discovery of new methodologies for the synthesis of carbasugars [38,39,40,41,42], the Li[NTf_2_]/tetraglyme (**[Li(G4)]TFSI**) system was found to promote a highly regio- and stereoselective ring opening of carbapyranoses 1,2-epoxides with an α-gluco configuration, such as those represented in Scheme 1. These substrates can be used as a pseudo glucosyl donor for the synthesis of C(1)-carbasugars featuring the challenging pseudo β-glucosidic bond.

## 2. Results and Discussion

**Scheme 2:**
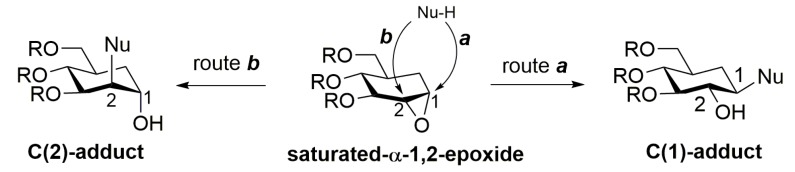
Possible nucleophilic addition pathways.

As has been extensively reported [43,44,45], carbapyranose 1,2-epoxides with a β-manno configuration can be opened successfully by *O*- and *N*-nucleophiles with excellent regio- and stereoselectivity through a sterically and electronically favored 1,2-trans-diaxial process. Conversely, carbapyranose 1,2-epoxides with an α-gluco configuration do not give such a good result both in terms of regioselectivity and yields. Indeed, in saturated-α-1,2-epoxides, both positions C(1) and C(2) present conflicting features for the smooth running of the reaction. An attack at C(1) is sterically and electronically favoured because the C(1) carbon is flanked by a small and relativity electron-rich methylene group, but leads to an unfavourable trans-diequatorial ring-opening (Scheme 2, route a), while attack at C(2), which could lead to a Fürst–Plattner favoured trans-diaxial ring-opening (Scheme 2, route b) is sterically hindered and electronically unfavourable. Nevertheless, a carbapyranose 1,2-epoxide with an α-gluco configuration turns out to be an effective β-carbaglucosyl donor for the synthesis of attractive pseudosaccharides and pseudodisaccharides, with a pseudo-gluco configuration of the carbapyranose unit [46].

**Scheme 3:**
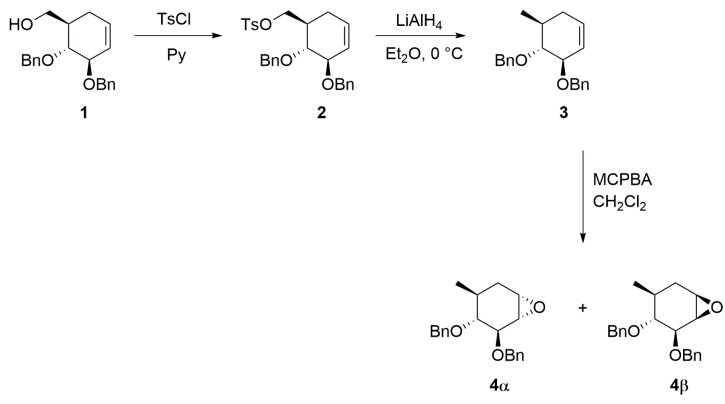
Preparation of α-gluco carbasugar 1,2-epoxides.

Our study started with the synthesis of the model substrate **4α** (Scheme 3) with the aim of investigating the possible effects of the Lewis acid partner on the regioselectivity of the ring opening reaction. Carbocyclic system **1** was built by the known Claisen rearrangement developed by Sudha and Nagarajan, starting from tri-*O*-acetyl glucal [47,48]. The primary hydroxyl group was then removed by means of a tosylation and a subsequent reduction with LiAlH_4_ in Et_2_O, to afford the desired methyl compound **3**. The latter was then subjected to epoxidation by means of m-chloroperoxybenzoic acid, to give the two diastereoisomeric epoxides **4α** and **4β**, characterized by α-gluco and β-manno configurations, respectively, in a 60:40 ratio due to the presence of the bulky benzylic ether on the C3 position.

Epoxide **4α** represents the model carbaglycosylating agent that was subjected to the ring opening reaction with either methanol or diacetone-D-galactose as nucleophiles in CH_2_Cl_2_, in the presence of a catalytic amount of Cu(OTf)_2_ as the Lewis acid catalyst (Table 1) [49].

As expected, no regioselectivity was observed in either case (Table 1, entry 1 and 2) and an almost 1:1 ratio of the products of the *anti* attack on C(1) and C(2) were isolated (a subsequent acetylation reaction afforded **5Ac**–**8Ac**, which allowed for the full characterization of the diastereoisomeric compounds).

Next, we employed LiClO_4_ as the Lewis acid and methanol in the dual role of solvent and nucleophile (Table 1, entry 3) [50].

**Figure 1:**
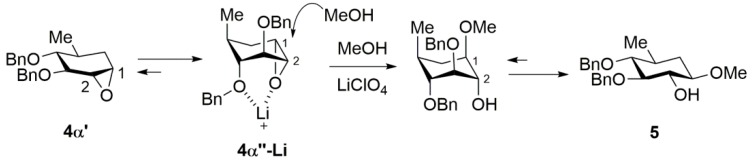
Conformational equilibrium shift due to the lithium coordination.

The reaction proceeded quite slowly (7 days, 80 °C), but, surprisigly, only one of the two possible regiosomeric products was isolated at the end of the reaction. It was possible to ascertain that only the C(1) attack occurred with complete regioselectivity. A working hypothesis to rationalise this result involves the presence of an ion with strong chelating properties, such as Li^+^, that is able to shift its ground state towards the less stable conformation **4α’’-Li**, in which the -OBn group on C(3) and the oxirane oxygen form a bidentate chelating system for the Li^+^ cation (Figure 1). This species can then undergo the favoured trans-diaxial ring-opening process with nucleophilic attack at C(1) to afford compound **5** as the only reaction product.

Although this was an astonishing result, it was very frustrating to verify that the reaction scope was limited to the use of nucleophiles in large excess, which clearly precludes the synthesis of complex structures. In addition, from a safety perspective, the use of the potentially explosive LiClO_4_ needs to be minimised.

Hence, having the experimental evidence that lithium salts can dramatically affect the regiochemical outcome of this reaction in hand, we searched the literature for alternatives. As mentioned above, SILs containing lithium salts, which are relatively polar solvents with Lewis acid characteristics, have been recently developed. Therefore, Li[NTf_2_], in either tri- or tetra-glyme form, **[Li(G3)]TFSI** or **[Li(G4)]TFSI**, respectively, were investigated.

In Table 2, the results of the ring-opening reaction of epoxide **4α**, using different SILs and methanol or diacetone-D-galactose as nucleophiles, are summarized.

All of the reactions were performed in an argon atmosphere after stirring the SILs under a high vacuum at 70 °C for at least 24 h, in order to remove traces of water that would have a detrimental effect on the reaction. In addition, the reaction temperature was set to 70 °C, which allowed for a decreased viscosity of the SILs and thus facilitated the reaction.

It was interesting to note that no conversion was observed with **[Li(G3)]TFSI** and methanol (20 equivalents) even after 3 days (entry 1), while in the case of **[Li(G4)]TFSI**, the epoxide-opening reaction did occur in 24 h under the same reaction conditions, even using 5 equivalents of methanol (entry 2). Indeed, the two SILs are characterized by a different size and slightly different hydrogen bond-donating ability (Kamlet–Taft α-values) [51], which could account for the results obtained. It is also worth mentioning that it is not unusual to observe a diverse outcome when these two SILs are employed under identical reaction conditions [25]. However, the number of reports concerning their use in synthesis is still limited and it is not possible yet to draw any general conclusion.

If this latter reaction is instead compared to the previous reaction with LiClO_4_ (Table 1, entry 3), two main aspects are to be highlighted: the shorter reaction time for the reaction to go to completion (24 h versus 8 days), and the lower regioselectivity, although it was still in favor of the C(1) attack (the regioisomeric α-methoxy alcohols **5** and **6** were found to be formed in a 60:40 ratio according to ^1^H-NMR on the crude versus complete regioselection). The effect on the reaction rate could be related to the higher amount of lithium, which is part of the solvent system, when SIL was employed. Concerning the second aspect, it has to be considered that Li^+^ in the SIL is already chelated by the glyme, which will result in a less coordinating cation than in the case of LiClO_4_. Therefore, it can be speculated that the bidentate chelate system will have a lower stability, and thus, a lower regioselectivity.

To further ascertain whether the presence of non-chelated lithium could enhance the regioselectivity, a new reaction was performed by adding additional LiTf_2_N (0.2 equivalents) to **[Li(G4)]TFSI** under otherwise identical conditions (entry 3). In this case, the only noticeable effect was a decrease in the reaction rate, while **5** and **6** were again obtained in a 60:40 ratio.

As a last attempt, we wanted to verify the reaction potential with a more synthetically useful nucleophile, an option that is ruled out when using LiClO_4_, as mentioned above. Therefore, the synthesis of a pseudodisaccharide was attempted, employing diacetone-D-galactose as the nucleophile (3.0 equivalents, entry 4). To our delight, after 24 h, we observed a high degree of regioselectivity with the attack of the more complex nucleophile having taken place at C1 (80:20) under the (partially) chelating conditions in **[Li(G4)]TFSI**. The regioselectivity was evaluated by HPLC-MS after acetylation of the crude mixture using compounds **7Ac** and **8Ac**, obtained in the copper catalyzed process, as standards. The HPLC chromatogram showed a regioisomeric ratio of 80:20 in favor of the C1 adduct **7Ac** (please refer to the ESI).

The increased regioselectivity that was observed, if compared with entry 2, seems to suggest that other features, beside the bidentate chelate system, are potentially involved in determining the stereo- and regiochemical outcome of the reaction. For instance, the steric hindrance of the nucleophile could play a major role in these outcomes.

Further studies are currently ongoing and aim to shed light on the optimal experimental conditions required to maximize the regioselectivity and elucidate the mechanism involved in the reaction. The results will be presented in due course.

## 3. Materials and Methods

All solvents and chemicals were used as purchased without further purification. SIL **[Li(G3)]TFSI** and **[Li(G3)]TFSI** were prepared following a previously reported procedure [30]. Chromatographic separations were performed on silica gel columns by flash (Kieselgel 40, 0.040–0.063 mm; Merck, Darmstadt, Germany) or by automated chromatography with Isolera^®^ Biotage, Uppsala, Sweden. Reactions were followed by thin-layer chromatography (TLC) on Merck aluminum silica gel (60 F254) sheets that were visualized under a UV lamp. Evaporation was performed in vacuo (rotating evaporator). Sodium sulfate was always used as the drying agent. Melting points were determined with a Kofler hot-stage apparatus and are uncorrected. Optical rotations were measured with a ATAGO AP-300 Automatic Polarimeter (Tokyo, Japan) at 25 °C. ^1^H- and ^13^C-NMR spectra were recorded with a Bruker Avance 250 MHz spectrometer (Billerica, MA, USA) using the indicated deuterated solvents. Chemical shifts are given in parts per million (ppm) (δ relative to the residual solvent peak for ^1^H and ^13^C). FTIR spectra were obtained with an IR Cary 600 FTIR (Agilent Technologies, Santa Clara, CA, USA). Yields refer to isolated and purified products. LC-MS evaluation and identification of the products was performed using a Shimadzu Nexera UHPLC chromatograph (Kyoto, Japan) equipped with a diode array detector and interfaced with an ABSciex API 3200 QTRAP mass spectrometer (Milan, Italy), using the following parameters. LC conditions: Column: Phenomenex Kinetex C18 EVO 3 × 150 mm, particle size 5 µm, flow 0.5 mL/min. Mobile phase A: ammonium formate 20 mM (pH 4.8) Mobile phase B: acetonitrile. Gradient: 95% A (1 min), then linear gradient to 100% B in 12 min, followed by a 3 min wash step at 100% B. MS conditions: Ionization mode: ESI. Curtain gas 10 mL/min; ion spray voltage: 5500 V; temperature: not used; declustering potential: 75 V; entrance potential: 10 V; collision energy: 50 eV; collision energy potential: 43 V. Elemental analyses were obtained using an Elementar Vario MICRO cube (Langenselbold, Germany). All the detailed experimental procedures, NMR and HPLC-MS spectra are given in the Supplementary Materials. 

## 4. Conclusions

In this work, we synthetized epoxide **4α** as a model of carbapyranose 1,2-epoxide with α-gluco configuration and we studied the corresponding nucleophilic ring-opening reaction under standard (Nu-H/CH_2_Cl_2_/Cu(OTf)_2_) or chelating conditions. It can be speculated that, when employing a Lewis acid such as LiClO_4_ in MeOH or, even more interestingly, when using an appropriate solvent such as **[Li(G4)]TFSI** containing an available Li^+^ cation, the conformational equilibrium of the starting epoxide can be altered, thus allowing for a highly (complete in the case of LiClO_4_) stereo- and C(1)-selective ring-opening process.

In brief, by using SIL **[Li(G4)]TFSI**, a new and efficient protocol for the synthesis of carbasaccharides or pseudodisaccharides with β-gluco configuration was revealed.

## Figures and Tables

**Table 1:**
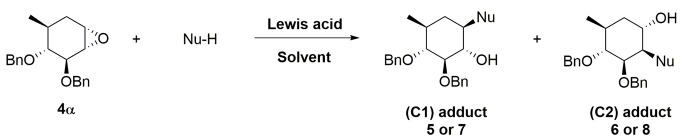
Lewis acid (L.A.)-mediated ring-opening addition of alcohol nucleophile (Nu-H) to epoxide **4α**.

Entry	Alcohol Nucleophile (Nu-H)	L.A.	Solvent	Time Temp.	Nu	C(1) Adduct^1^ (%)	C(2) Adduct^1^ (%)	Isolated Yield^2^
1	MeOH	Cu(OTf)_2_	CH_2_Cl_2_	12 h r.t.	OMe	**5** (50)	**6** (50)	C1: 32% C2: 32%
2	1,2:3,4-di-*O*-isopropylidene-d-galactopyranose	Cu(OTf)_2_	CH_2_Cl_2_	12 h r.t.	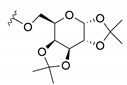	**7** (55)	**8** (45)	C1: 35% C2: 28%
3	MeOH	LiClO_4_	MeOH	7 days 80°C	OMe	**5** (>99)	/	C1: 55%

^1^ regioselectivity estimated by ^1^H-NMR of the crude reaction product. ^2^ yields calculated on the corresponding acetyl derivatives **5Ac**–**8Ac** that were easily separable.

**Table 2:**
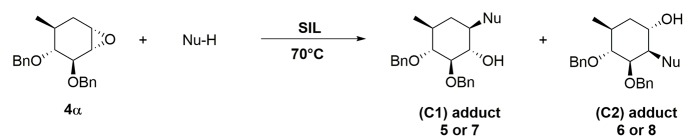
Results for the ring-opening addition of alcohol nucleophiles to epoxide **4α** in various SILs and different conditions.

Entry	Alcohol Nucleophile (Nu-H)	SIL	Conditions	Nu	C(1) Adduct (%)	C(2) Adduct (%)
1	MeOH	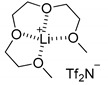 **[Li(G3)]TFSI**	i) IL dried ii) 70 °C 3 days	OMe	/	/
2	MeOH	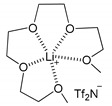 **[Li(G4)]TFSI**	i) IL dried ii) 70 °C 24 h	OMe	**5** (60)^1^	**6** (40)^1^
3	MeOH	**[Li(G4)]TFSI**	i) IL dried ii) 70 °C LiTf_2_N excess 3 days	OMe	**5** (60)^1^	**6** (40)^1^
4	1,2:3,4-di-*O*-isopropylidene-d-galactopyranose	**[Li(G4)]TFSI**	i) IL dried ii) 70 °C 24 h	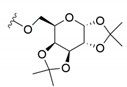	**7Ac** (80)^2^	**8Ac** (20)^2^

^1^ regioselectivity estimated by ^1^H-NMR of the crude reaction product. ^2^ regioselectivity assessed by HPLC-MS of the acetylation crude, using **7Ac** and **8Ac** previously isolated as standards.

## Supplementary Materials

The following are available online, all the detailed experimental procedures, NMR and HPLC-MS spectra.
