# Supplementary material for: Remarkable Effect of [Li(G4)]TFSI Solvate Ionic Liquid (SIL) on the Regio- and Stereoselective Ring Opening of α-Gluco Carbasugar 1,2-Epoxides
Source: Molecules. 2019 Aug 14;24(16):2946. doi: 10.3390/molecules24162946 (PMC6720504; doi:10.3390/molecules24162946)
Supplement: Supplementary file 1 [file molecules-24-02946-s001.pdf]

Supporting Information

Remarkable effect of [Li(G4)]TFSI solvate ionic liquid (SIL) on the regio- and stereoselective ring opening of α-gluco carbasugar 1,2-epoxides

Sebastiano Di Pietro[1], Vittorio Bordoni[1,a], Andrea Mezzetta[1], Cinzia Chiappe[#], Giovanni Signore[2], Lorenzo Guazzelli[1,*], Valeria Di Bussolo[3,*]

[1] Dipartimento di Farmacia, Università di Pisa, Via Bonanno 33, 56126 Pisa, Italy; sebastiano.dipietro@unipi.it (S.D.P.); andrea.mezzetta@for.unipi.it (A.M.)
[a] Present address: Max Planck Institute of Colloids and Interfaces, Am Mühlen- berg 1, 14476 Potsdam (Germany)
[2] Fondazione Pisana per la Scienza, via F. Giovannini 13, 56017, San Giuliano Terme (PI), Italy; g.signore@fpscience.it (G.S.)
[3] Dipartimento di Chimica e Chimica Industriale, Università di Pisa, Via G. Moruzzi 3, 56124 Pisa, Italy
* Correspondence: valeria.dibussolo@unipi.it (V.D.B.); lorenzo.guazzelli@unipi.it (L.G.)
\# in beloved memory

Contents

Experimental procedures, characterization data, NMR and HPLC-MS spectra.

3,4-Di-*O*-benzyl-6-*O*-tosyl-5a-carba-D-glucal (2). A solution of primary alcohol **1** (0.244 mg, 0.753 mmol, 1 eq) in anhydrous pyridine (2.6 mL) was added to solid tosyl chloride (215.3 mg, 1.13 mmol, 1.5 eq) at 0 °C and the reaction mixture was allowed to warm up to room temperature in 24 h. Then, diethylether was added to the mixture, which was washed with distilled water, ice and diluted HCl until pH<7. Afterwards, it was washed with saturated aqueous solution of $NaHCO_3$ and brine until pH>7 and the organic layer was dried with anhydrous magnesium sulfate and the solvent evaporated. Compound **2** (252 mg, yield = 70%), was used without any further purification. R_f = 0.24 (8:2 hexane/AcOEt); $[\alpha]_D^{20}$ = -2.5 (*c* 1.26, $CHCl_3$). FTIR (neat film) ν (cm^{-1}) = 3031, 2903, 1359, 1175, 1094, 926, 813, 666. ^{1}H-NMR (250 MHz, $CDCl_3$) δ (ppm) = 7.75 (d, 2H, *J* = 8.3 Hz), 7.35-7.27 (m, 10H), 7.21-7.15 (m, 2H), 5.76-5.63 (m, 2H), 4.84 (d, 1H, *J* = 10.9 Hz), 4.67 (d, 1H, *J* = 11.6 Hz), 4.60 (d, 1H, *J* = 11.6 Hz), 4.48 (d, 1H, *J* = 10.9 Hz), 4.21 (dd, 1H, *J* = 9.2, 5.2 Hz), 4.17-4.09 (m, 2H), 3.56 (dd, 1H, *J* = 9.7, 7.1 Hz), 2.41 (s, 3H), 2.26-2.07 (m, 3H). ^{13}C-NMR (62.5 MHz, $CDCl_3$) δ (ppm) = 145.0, 138.5, 130.0, 128.6, 128.1, 128.0, 126.3, 80.9, 78.6, 74.4, 71.5, 70.7, 38.4, 28.0, 21.8.

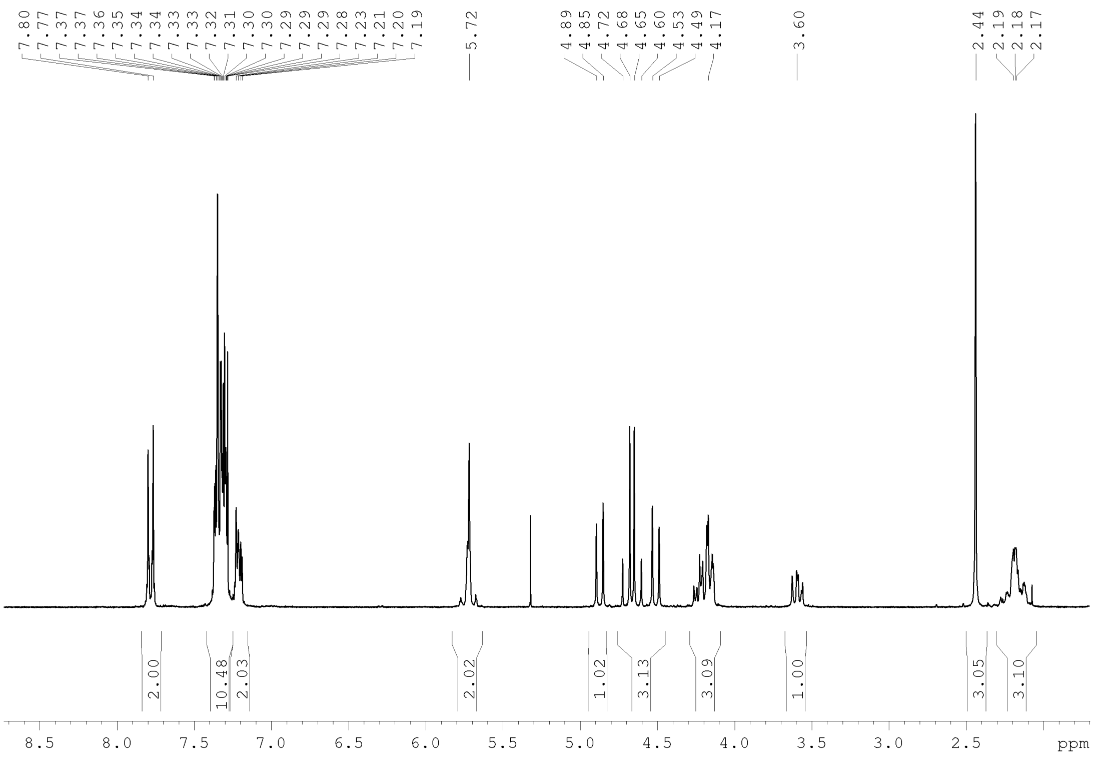

Figure S 1. ^{1}H-NMR spectrum compound 2.

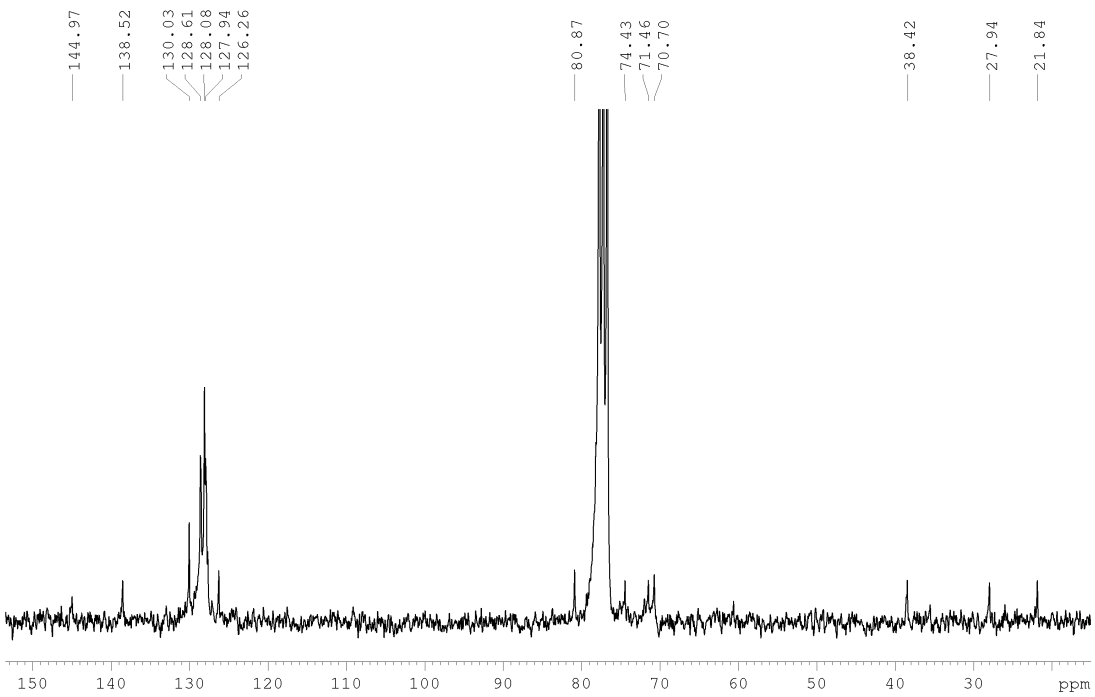

Figure S 2. ^{13}C-NMR spectrum of compound 2.

3,4-di-*O*-benzyl-6-Deoxy-5a-carba-D-glucal (3). To a solution of tosylate **2** (252 mg, 0.53 mmol, 1 eq) in anhydrous Et_2O (22 mL) were added 176 mg of lithium aluminium hydride (4.639 mmol, 8.8 eq) at 0 °C. The reaction mixture was stirred for 4 hours, then it was diluted with Et_2O and ice and a 10% aqueous sodium hydroxide was added dropwise until the formation of a white precipitate. The two phases were separated and the organic one was dried with Na_2SO_4 and filtered; after the evaporation of the solvent the methyl-substituted olefin **3** (156 mg, yield = 96%) was obtained as a yellow oil and used without further purification. R_f = 0.49 (8:2 hexane/AcOEt); $[\alpha]_D^{20}$ = +10.2 (*c* 0.16, $CHCl_3$). FTIR (neat film) ν (cm^{-1}) = 3064, 3030, 2951, 2888, 1496, 1454, 1101,735, 696. ^{1}H-NMR (250 MHz, $CDCl_3$) δ (ppm) = 7.42-7.27 (m, 10H), 5.81-5.62 (m, 2H), 4.93 (d, 1H, *J* = 11.0 Hz), 4.70 (s, 2H), 4.67 (d, 1H, *J* = 11.0 Hz), 4.21-4.13 (m,1H), 3.32 (dd, 1H, *J* = 10.6, 7.6 Hz), 2.20-2.14 (m, 1H), 1.95-1.76 (m, 2H), 1. 09 (d, 3H, *J* = 6.2 Hz). ^{13}C-NMR (62.5 MHz, $CDCl_3$) δ (ppm) = 132.7, 128.7, 128.6, 128.2, 128.1, 127.9, 127.7, 119.1, 75.0, 74.0, 53.7, 42.6, 34.2, 27.5, 14.5.

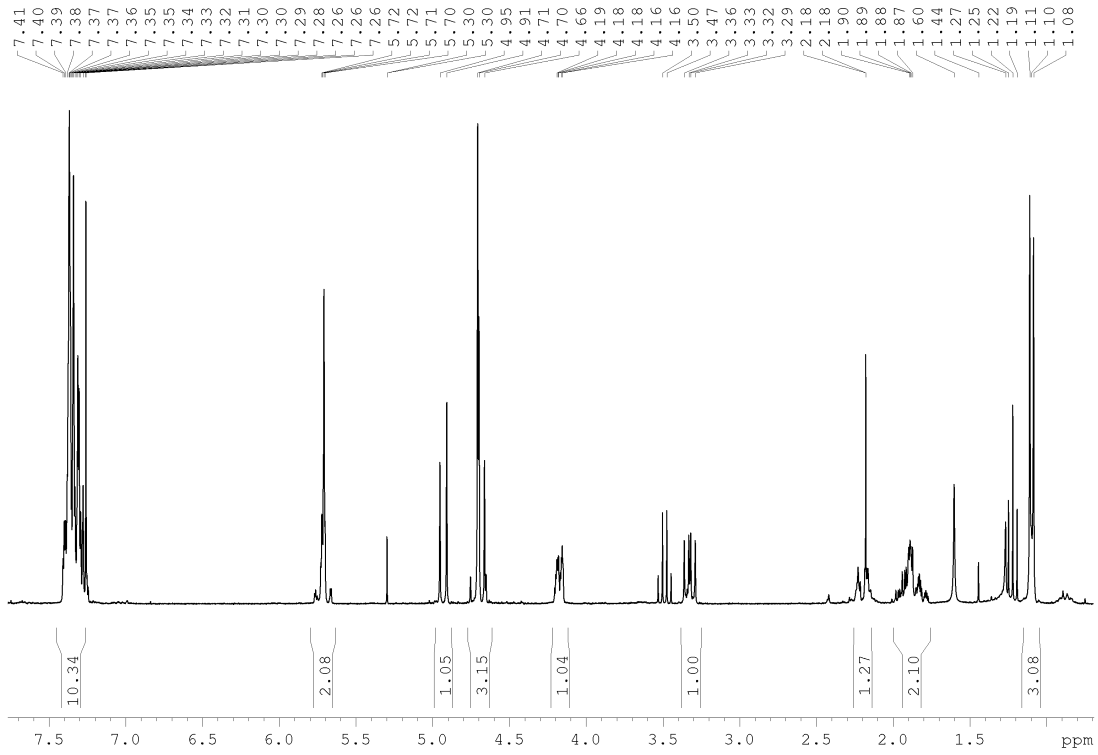

Figure S 3. ^{1}H-NMR spectrum of compound 3.

Reaction of methyl-substituted olefin 3 with MCPBA (4α and 4β). 131 mg of methyl-substituted olefin **3** (0.425 mmol, 1 eq) were solubilized in anhydrous dichloromethane (13 mL) and 146.8 mg of *m*-chloro perbenzoic acid were added (0.851 mmol, 2 eq) at 0 °C. The reaction mixture was stirred at room temperature for 24 h. Then, more dichloromethane was added and the organic phase was washed respectively with a 10% aqueous Na_2SO_4 solution, aqueous saturated solution of $NaHCO_3$ and brine. The organic layer was dried with anhydrous sodium sulfate and the solvent evaporated. The crude diasteroisomeric mixture of epoxides **4α** and **4β** was purified by flash chromatography (SiO_2, hexane : ethyl acetate = 9:1) to afford epoxides **4α** (58 mg) and **4β** (12 mg) as white solids. **3,4-Di-*O*-benzyl-6-deoxy-1,2-anhydro-5a-carba-α-D-glucopyranose (4α).** M.p. 62-65°C; R_f = 0.40 (8:2 hexane/AcOEt); $[\alpha]_D^{20}$ = +6.8 (*c* 2.17, $CHCl_3$). ^{1}H-NMR (250 MHz, $CDCl_3$) δ (ppm) = 7.39-7.22 (m, 10H), 4.79 (d, 1H, *J* = 11.0 Hz), 4.76 (d, 1H, *J* = 11.3 Hz), 4.65 (d, 1H, *J* = 11.3 Hz), 4.54 (d, 1H, *J* = 11.0 Hz), 3.70 (d, 1H, *J* = 7.9 Hz), 3.15-3.09 (m,2H), 2.96 (dd, 3H, *J* = 10.8, 7.9 Hz), 2.22-2.10 (m, 1H), 1.66-1.39 (m, 2H), 0.93 (d, 3H, *J* = 6.3 Hz). ^{13}C-NMR (62.5 MHz, $CDCl_3$) δ (ppm) = 138.9, 138.9, 128.7,

128.5, 128.1, 127.7, 84.5, 80.6, 74.8, 72.8, 54.5, 52.9, 33.1, 27.5, 17.8. Anal. Calcd for $C_{21}H_{24}O_3$: C, 77.75; H, 7.46. Found: C, 77.25; H, 7.32. **3,4-Di-*O*-benzyl-6-deoxy-1,2-anhydro-5a-carba-β-D-mannopyranose (4β).** M.p. 28-31 °C; R_f = 0.26 (8:2 hexane/AcOEt); $[\alpha]_D^{20}$ = +14.9 (*c* 0.99, $CHCl_3$). ^{1}H-NMR (250 MHz, $CDCl_3$) δ (ppm) = 7.39-7.27 (m, 10H), 4.88 (d, 1H, *J* = 10.7 Hz), 4.83 (s, 2H), 4.58 (d, 1H, *J* =10.7 Hz), 3.80 (dd, 1H, *J* = 8.2, 1.9 Hz), 3.36-3.27 (m, 2H), 3.24-3.19 (m, 1H), 2.07(dd, 1H, *J* = 5.0 Hz), 1.71-1.55 (m,3H), 0.98 (d, 3H, *J* = 6.0 Hz). ^{13}C-NMR (62.5 MHz, $CDCl_3$) δ (ppm) = 138.8, 138.7, 128.6, 128.3, 128.1, 127.8, 82.3, 81.7, 75.7, 72.6, 55.9, 53.8, 34.7, 32.2, 17.7. Anal. Calcd for $C_{21}H_{24}O_3$: C, 77.75; H, 7.46. Found: C, 77.32; H, 7.38.

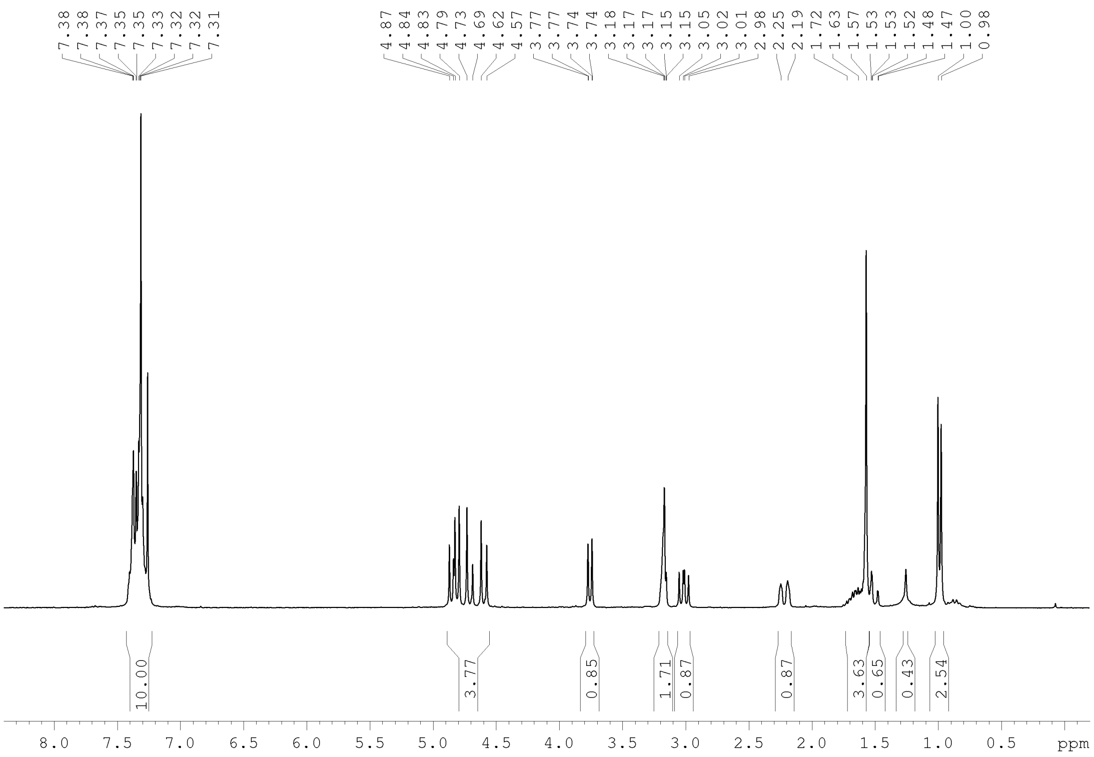

Figure S 4. ^{1}H-NMR spectrum of compound 4α.

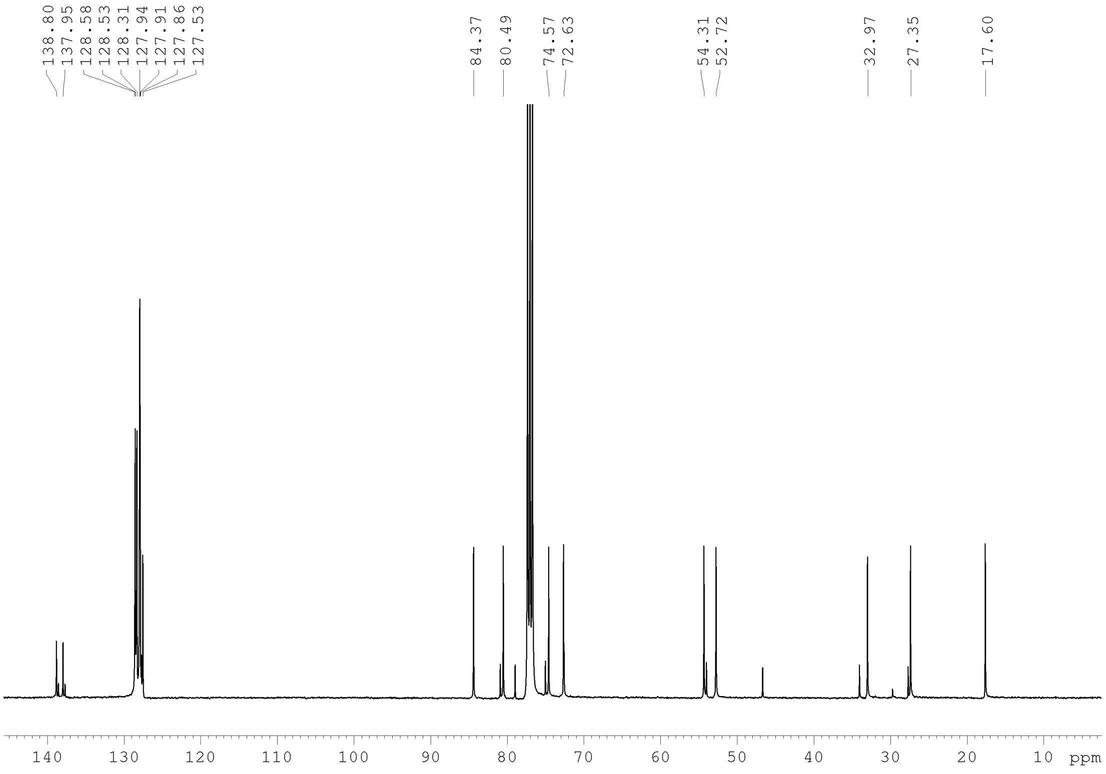

Figure S 5. ^{13}C-NMR spectrum of compound 4α.

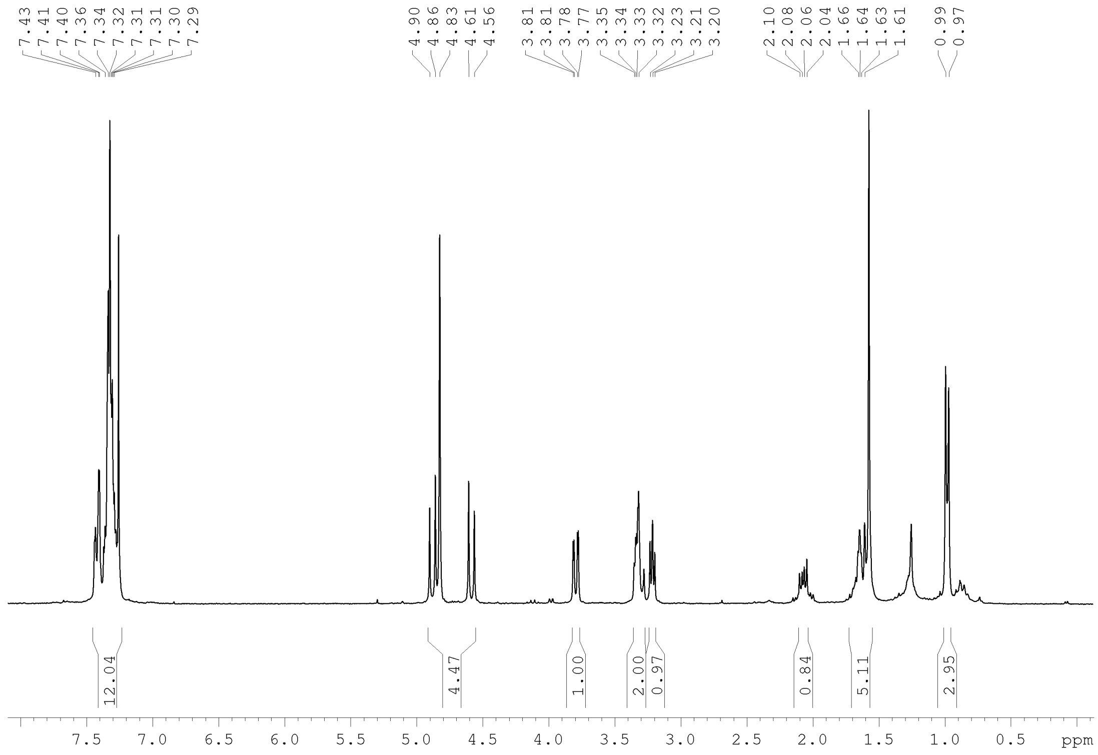

Figure S 6. ^{1}H-NMR spectrum of compound 4β.

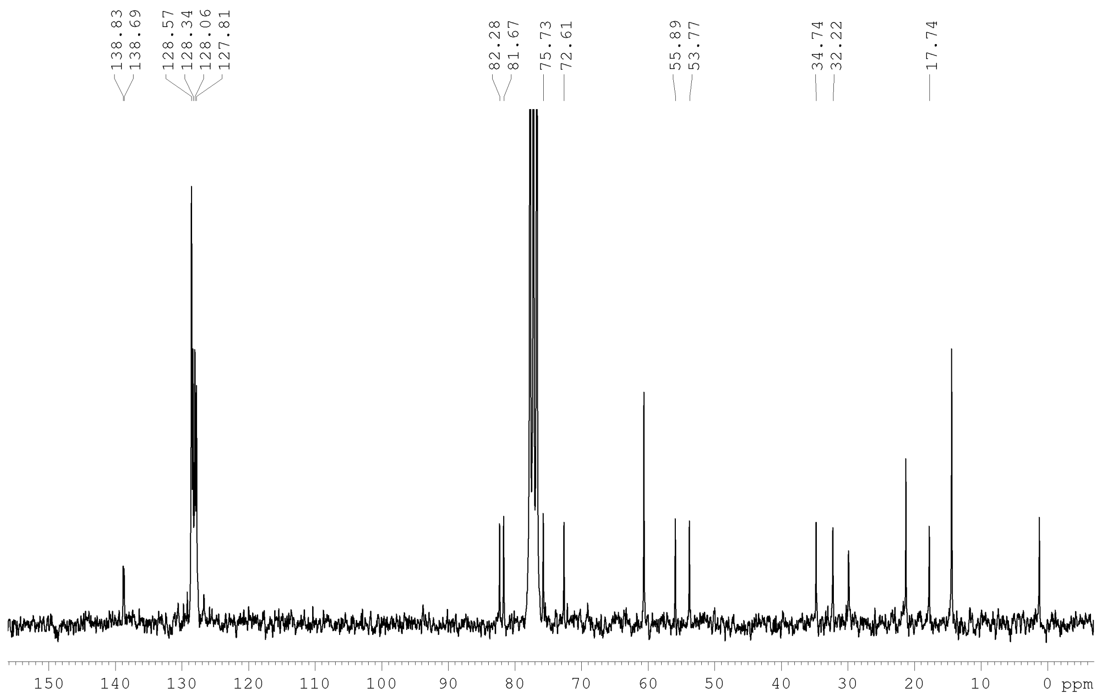

Figure S 7. ^{13}C-NMR spectrum of compound 4β.

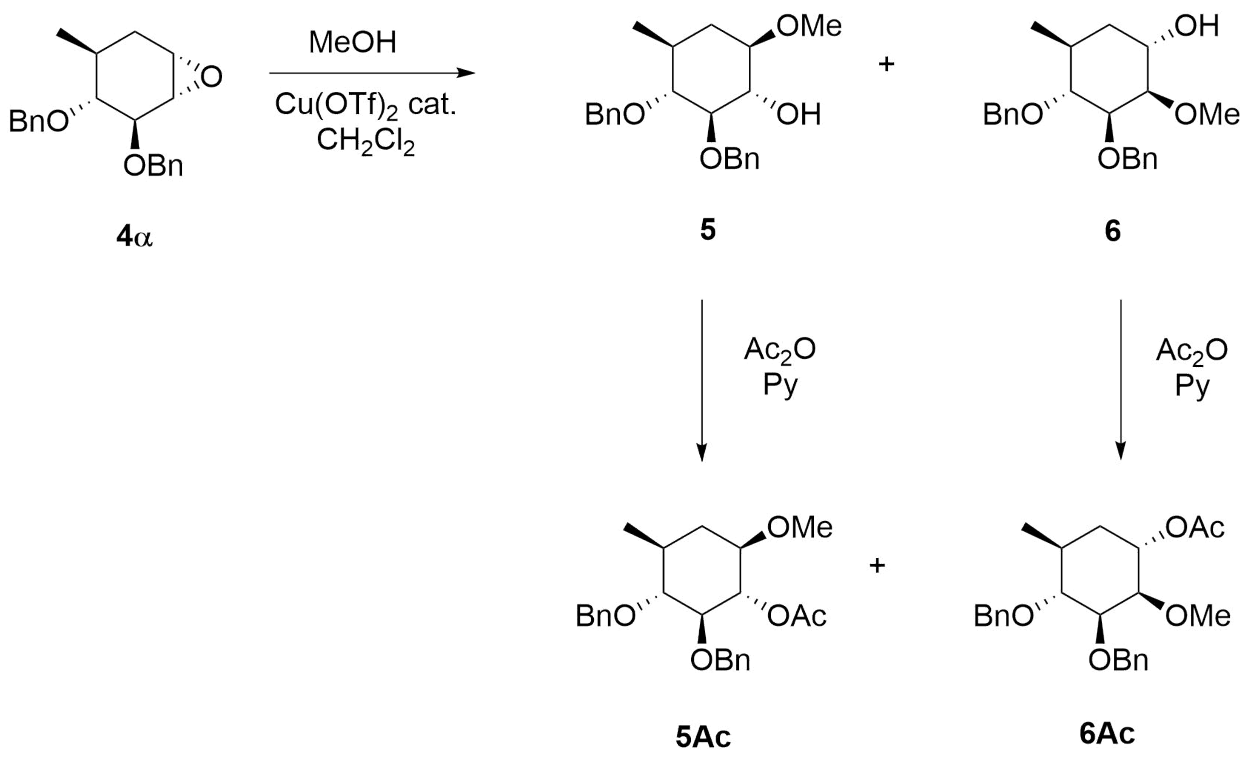

Scheme S1. Cu(OTf)2-catalyzed methanolysis of methyl-substituted epoxide 4α.

Reaction of epoxide 4α with $Cu(OTf)_2$/MeOH/CH_2Cl_2. A solution of epoxide 4**α** (0.022 g, 0.067 mmol) in anhydrous dichloromethane (0.2 mL) was treated with MeOH (10.8 mL, 0.268 mmol, 4.0 eq) and $Cu(OTf)_2$ (0.007 g, 0.020 mmol, 0.3 eq) and the resulting reaction mixture was stirred for 48 h at room temperature. After dilution with dichloromethane, the organic phase was washed with a saturated aqueous $NaHCO_3$ and brine. The solvent was evaporated to afford a crude product (0.022 g, 99% yield) consisting of a 1:1 mixture of the two regioisomeric *trans*-methoxy alcohols **5** and **6** (by ^{1}H-NMR) impossible to resolve. Then, a solution of the 1:1 mixture of *trans*-methoxy alcohols **5** and **6** in anhydrous pyridine (0.2 mL) was treated with acetic anhydride (0.1 mL) at 0 °C. The reaction mixture was stirred for 18 h at room temperature and co-evaporated with toluene afforded a 1:1 mixture of the corresponding acetyl derivatives **5Ac** and **6Ac**, which was purified by preparative TLC using a 2:7:1 hexane/CH_2Cl_2/(*i*-Pr)$_2$O mixture as eluent to afford pure methoxy derivates **5Ac** (0.008 g, yield = 32%) and **6Ac** (0.008 g, yield = 32%) respectively as colorless liquids. **2-*O*-Acetyl-3,4-di-*O*-benzyl-6-deoxy-1-*O*-methyl-5a-carba-β-D-glucopyranoside (5Ac).** R_f = 0.31 [2:7:1 hexane/CH_2Cl_2/(*i*-Pr)$_2$O]; $[\alpha]_D^{20}$ = +22.8 (*c* 0.72, $CHCl_3$). ^{1}H-NMR (250 MHz, $CDCl_3$) δ (ppm) = 7.37-7.27 (m, 10H), 5.02 (t, 1H, J = 9.6 Hz), 4.86 (d, 1H, J = 10.7 Hz), 4.82 (d,1H, J = 11.3 Hz), 4.65 (d, 1H, J = 11.3 Hz),

4.59 (d, 1H, J = 10.7 Hz), 3.46 (t, 1H, J = 9.6Hz), 3.33 (s, 3H), 3.25-3.16 (m, 1H), 3.12 (dd, 1H, J = 10.2, 9.6 Hz), 2.10-1.99 (m, 1H), 1.97 (s, 3H), 1.65-1.50 (m, 2H), 1.07 (d, 3H, J = 6.3 Hz). ^{13}C-NMR (62.5 MHz, $CDCl_3$) δ (ppm) = 170.5, 138.7, 138.5, 128.6, 128.3, 127.9, 127.8, 86.2, 84.2, 79.1, 75.7, 75.6, 57.4, 34.3, 33.4, 21.3, 18.3. Anal. Calcd for $C_{24}H_{30}O_5$: C, 72.34; H, 7.59. Found: C, 72.25; H, 7.45. **1-*O*-Acetyl-3,4-di-*O*-benzyl-6-deoxy-2-*O*-methyl-5a-carba-α-D-mannopyranoside (6Ac).** R_f = 0.52 [2:7:1 hexane/CH_2Cl_2/(i-Pr)$_2$O]; $[\alpha]_D^{20}$ = +35.6 (c 0.74, $CHCl_3$). ^{1}H-NMR (250 MHz, $CDCl_3$) δ (ppm) =7.41-7.27 (m, 10H), 5.07-5.00 (m, 1H), 4.96 (d, 1H, J = 10.5 Hz), 4.76 (d, 1H, J = 12.0 Hz), 4.67 (d, 1H, J = 12.0 Hz), 4.60 (d, 1H, J = 10.5 Hz), 3.63 (dd, 1H, J = 9.6, 3.0Hz), 3.51-3.46 (m, 1H), 3.48 (s, 3H), 3.38 (t, 1H, J = 9.6 Hz), 1.96 (s, 3H), 1.88-1.72 (m,1H), 1.66-1.61 (m, 2H), 1.04 (d, 3H, J = 6.3 Hz). ^{13}C-NMR (62.5 MHz, $CDCl_3$) δ (ppm) = 170.0, 139.1, 138.7, 128.6, 128.3, 128.2, 127.9, 127.7, 83.2, 81.3, 78.4, 75.9, 73.0, 69.2, 59.2, 32.5, 32.0, 21.3, 18.2. Anal. Calcd for $C_{24}H_{30}O_5$: C, 72.34; H, 7.59. Found: C, 72.29; H, 7.39.

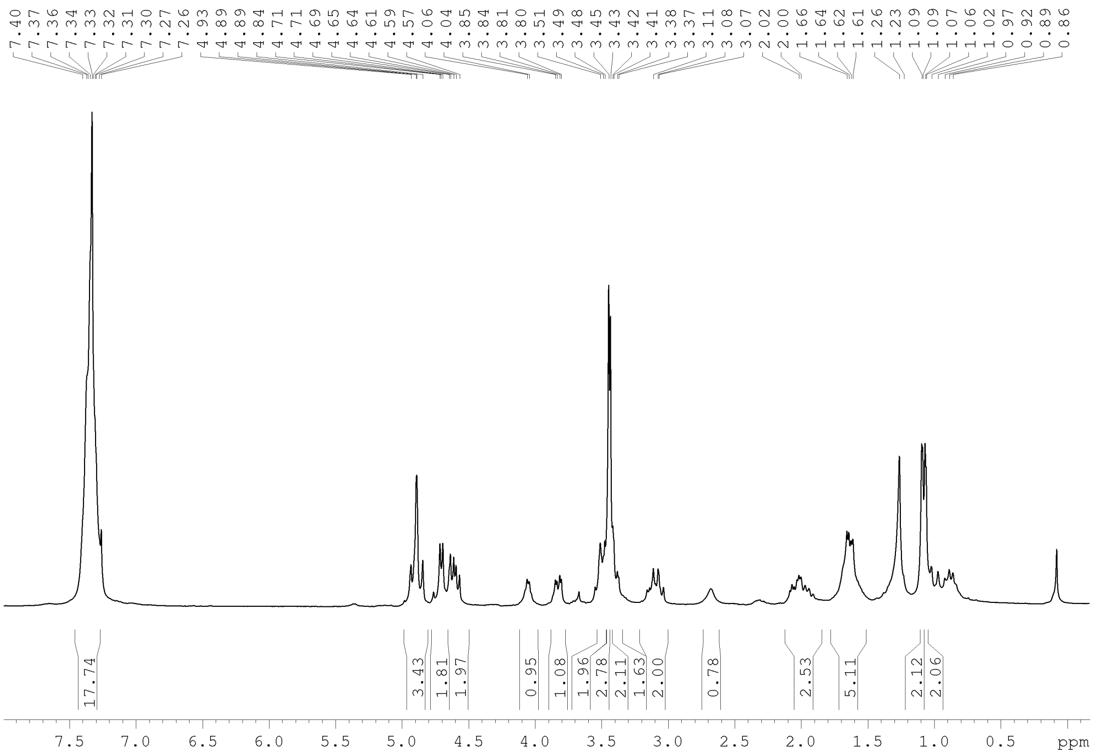

Figure S 8. ^{1}H-NMR spectrum of the 5-6 mixture.

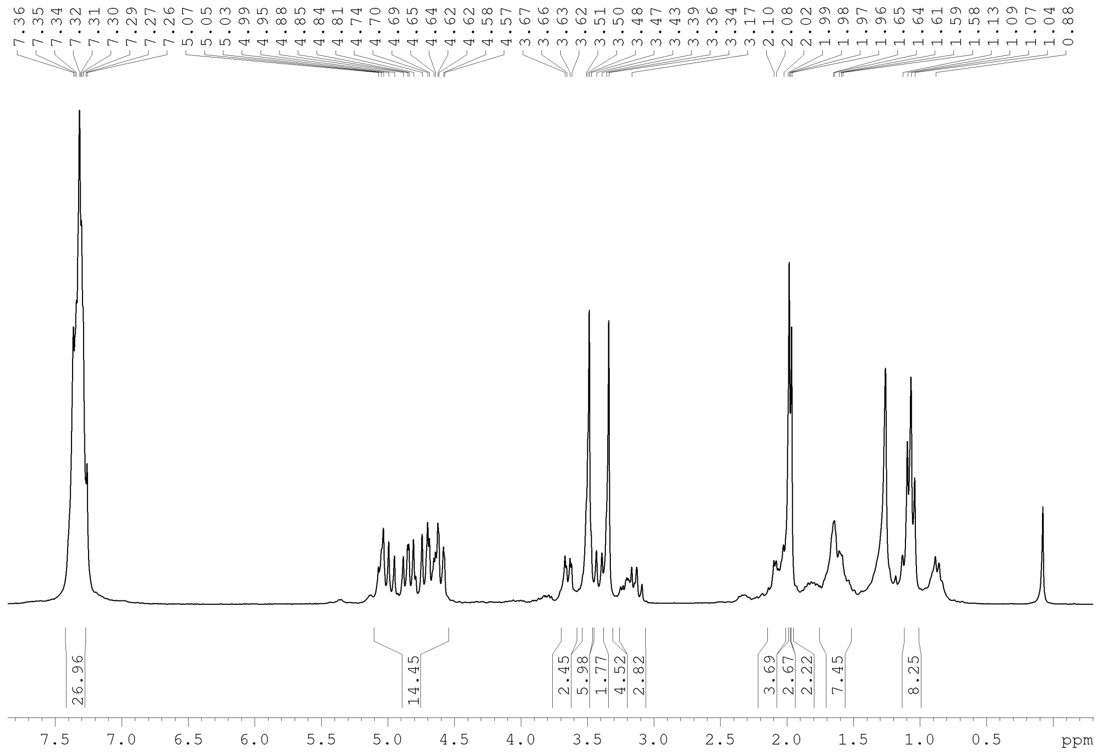

Figure S 9. ^{1}H-NMR spectrum of the 5Ac-6Ac mixture.

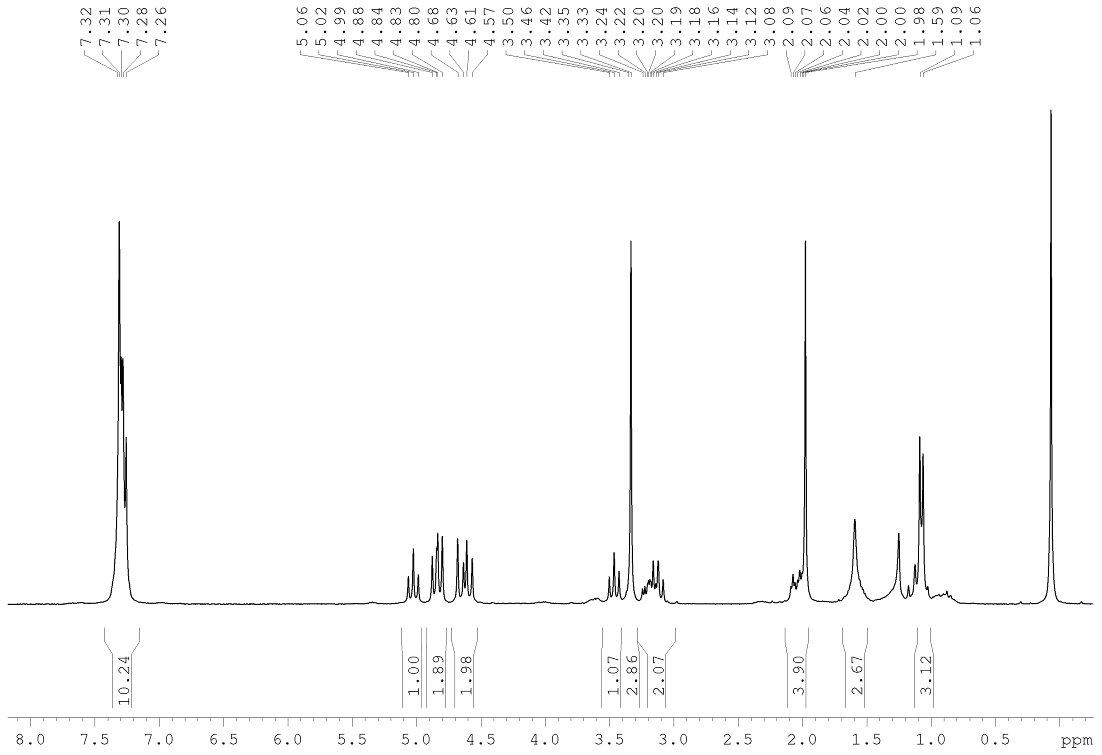

Figure S 10. ^{1}H-NMR spectrum of compound 5Ac.

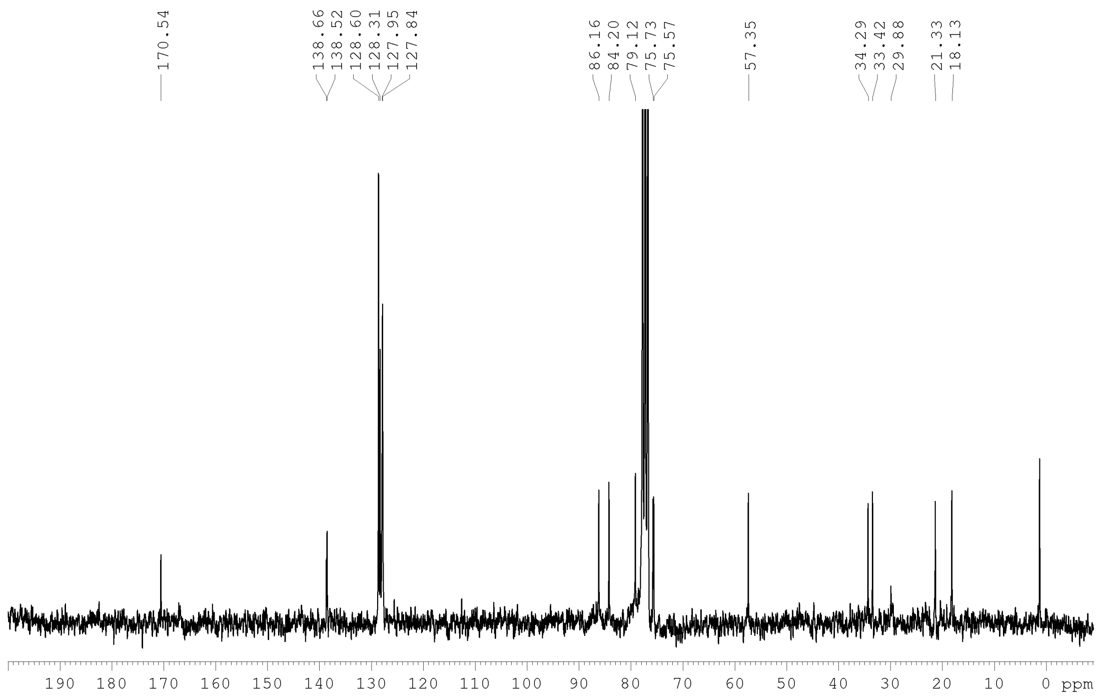

Figure S 11. ^{13}C-NMR spectrum of compound 5Ac.

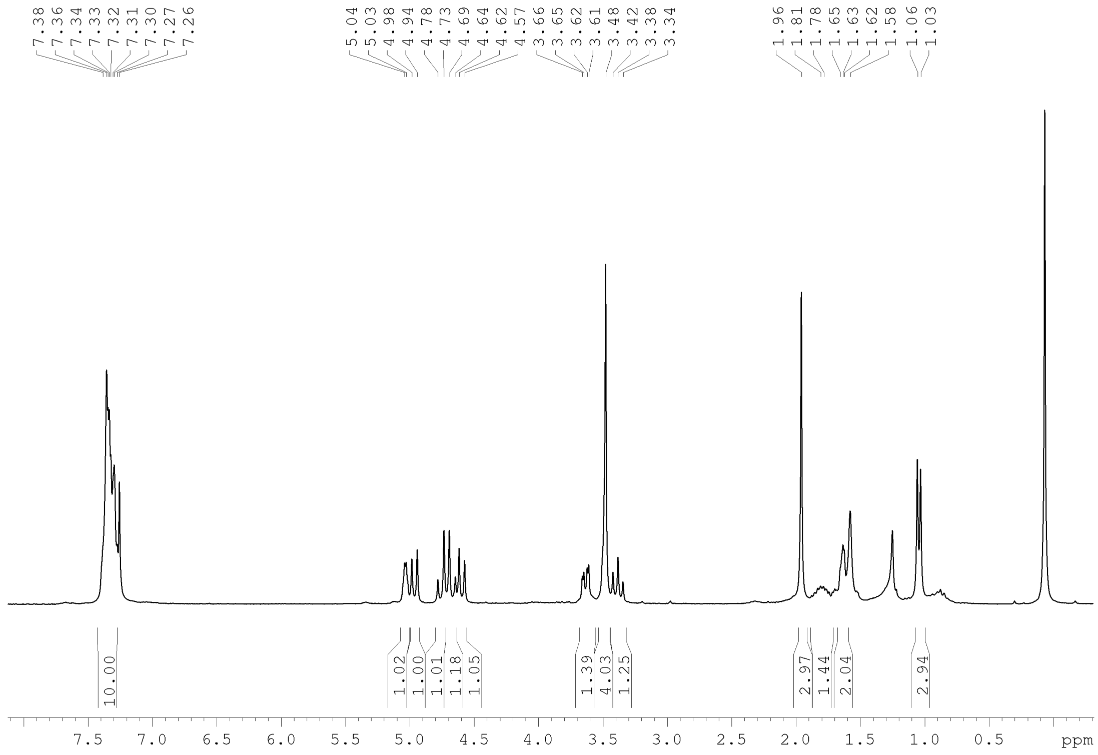

Figure S 12. ^{1}H-NMR spectrum of compound 6Ac.

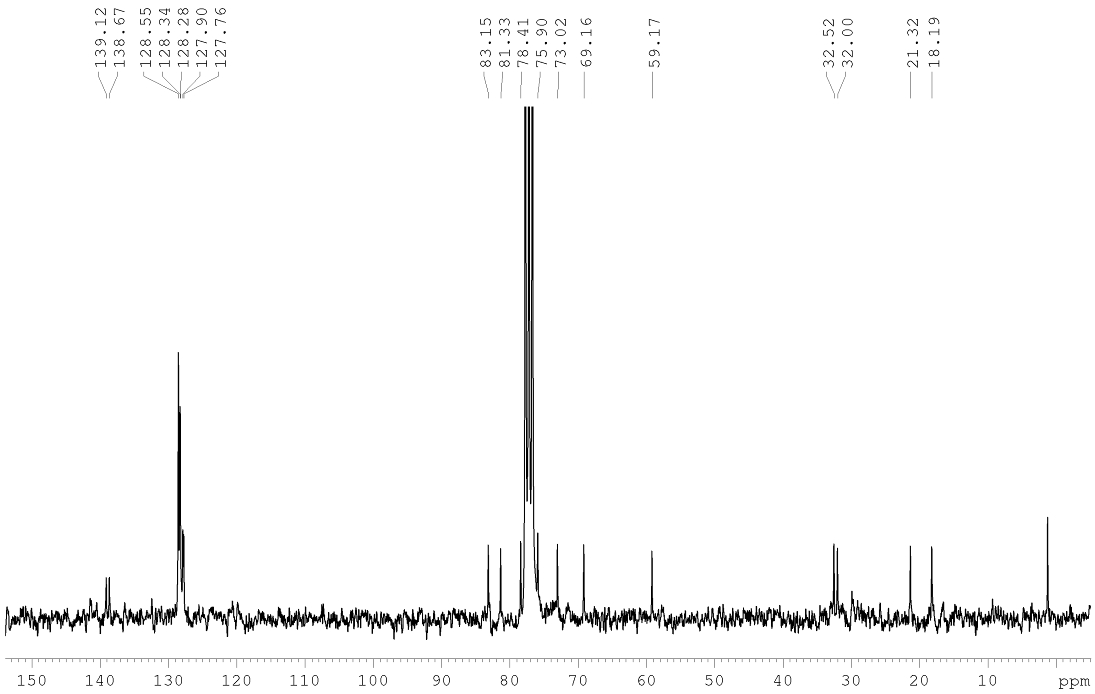

Figure S 13. ^{13}C-NMR spectrum of compound 6Ac.

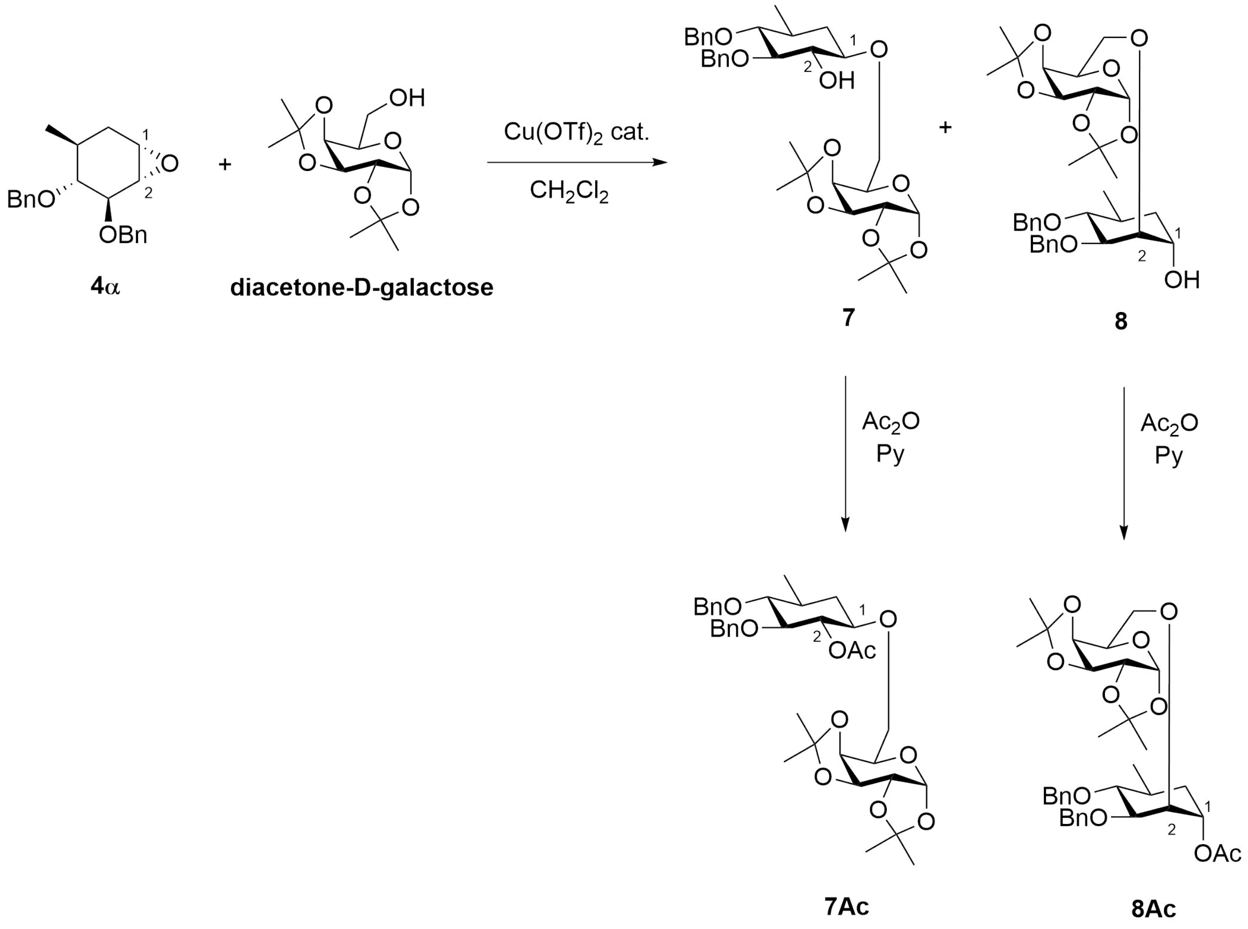

Scheme S2. Cu(OTf)2-catalyzed addition of diacetone-D-galactose to epoxide 4α

Reaction of epoxide 4α with diaceton-D-galactose/Cu(OTf)$_2$/CH$_2$Cl$_2$. Epoxide **4α** (50.9 mg, 0.157 mmol, 1 eq) was dissolved in anhydrous dichloromethane (0.75 mL) and it was added dropwise to a solution of diaceton-D-galactose (0.471 mmol, 3 eq) in dichloromethane (0.75 mL). Then, Cu(OTf)$_2$ (22.71 mg, 0.0628 mmol, 0.4 eq) is added to the reaction mixture, which was stirred for 64 h at room temperature. The crude product, consisting of the two regioisomer **7** and **8** in a 55:45 ratio (^{1}H-NMR), was purified by preparative TLC (SiO$_2$, eluent CH$_2$Cl$_2$:(*i*-Pr)$_2$O = 8:2) to afford the two products, which were successively acetylated in order to evaluate their regiochemistry. **1,2;3,4-*O*-Diisopropylidene-6-*O*-(3,4-di-*O*-benzyl-6-deoxy-5a-carba-β-D-1-glucosyl)-α-D-galactopyranoside (7).** 32.4 mg, yield = 35%, R_f = 0.32 (8:2 CH$_2$Cl$_2$/(*i*-Pr)$_2$O); $[\alpha]_D^{20}$ = -34.4° (*c* 1.75, CHCl$_3$); FTIR (neat film) ν (cm^{-1}) = 3565, 2927, 2359, 2340, 1733, 1454, 1259, 1067, 803. ^{1}H-NMR (250 MHz, CDCl$_3$) δ (ppm) = 7.42-7.27 (m, 10H), 5.56 (d, 1H, *J* = 5.0 Hz), 4.91 (dt, 3H, *J* = 20.6, 11.1 Hz), 4.63-4.55 (m, 2H), 4.32 (dd, 1H, *J* = 5.0, 2.4 Hz), 4.24 (dd, 1H, *J* = 7.9, 1.9 Hz), 4.01-3.94 (m, 1H), 3.86 (dd, 1H, *J* = 10.5, 4.8 Hz), 3.65-3.54 (m, 2H),

3.43 (t, 1H, J = 9.1 Hz), 3.33-3.21 (m, 1H), 3.03 (dd, 1H, J = 10.4, 8.9 Hz), 1.96 (dt, 1H, J = 13.2, 3.9 Hz), 1.65-1.55 (m, 1H), 1.54 (s, 1H), 1.45 (s, 1H), 1.33 (s, 1H), 1.25-1.09 (m, 1H), 1.05 (d, 1H, J = 6.5 Hz). ^{13}C-NMR (62.5 MHz, $CDCl_3$) δ (ppm) = 139.3, 138.8, 128.5, 128.5, 128.2, 128.1, 127.7, 127.6, 109.5, 108.8, 96.5, 85.9, 85.8, 81.4, 77.4, 75.6, 75.4, 71.3, 70.9, 70.7, 68.7, 67.6, 34.8, 33.6, 26.2, 26.1, 25.1, 24.5, 18.3. Anal. Calcd for $C_{33}H_{44}O_9$: C, 67.79; H, 7.59. Found: C, 67.68; H, 7.47. **1,2;3,4-*O*-Diisopropylidene-6-*O*-(3,4-di-*O*-benzyl-6-deoxy-5a-carba-α-D-2-mannosyl)-α-D-galactopyranoside (8).** 25 mg, yield = 28%, R_f = 0.4 (8:2 CH_2Cl_2/(*i*-Pr)$_2$O); $[\alpha]_D^{20}$ = -35.5 (*c* 1.41, $CHCl_3$); FTIR (neat film) ν (cm^{-1}) = 3529, 2960, 2926, 2359, 2340, 1733, 1376, 1095, 1064, 1015, 803. ^{1}H-NMR (250 MHz, $CDCl_3$) δ (ppm) = 7.36-7.23 (m,10H), 5.52 (d, 1H, J = 5.0 Hz), 4.73-4.50 (m, 5H), 4.36-4.28 (m, 2H), 4.15-3.96 (m, 2H), 3.87-3.77 (m, 2H), 3.67 (d, 1H, J = 8.2 Hz), 3.63-3.56 (m, 1H), 3.45 (t, 1H, J = 6.7 Hz), 2.07-1.99 (m, 1H), 1.69 (t, 2H, J = 5.8 Hz), 1.54 (s, 3H), 1.44 (s, 3H), 1.33 (s, 6H), 1.08 (d, 3H, J = 7.0 Hz). ^{13}C-NMR (62.5 MHz, $CDCl_3$) δ (ppm) = 139.0, 138.9, 128.5, 128.4, 127.9, 127.8, 127.6, 127.6, 109.3, 108.7, 96.4, 81.8, 81.6, 79.6, 73.7, 73.0, 70.9, 70.7, 70.6, 69.9, 66.9, 66.0, 34.7, 31.7, 26.2, 26.1, 25.0, 24.6, 18.2. Anal. Calcd for $C_{33}H_{44}O_9$: C, 67.79; H, 7.59. Found: C, 67.75; H, 7.50

1,2;3,4-*O*-Diisopropylidene-6-*O*-(2-acetyl-3,4-di-*O*-benzyl-6-deoxy-5a-carba-β-D-1-glucosyl)-α-D-galactopyranoside (7Ac). To solution of pseudodisaccharide **7** in anhydrous pyridine (0.1 mL) were added at 0 °C 0.05 ml of acetic anhydride and the reaction was stirred for 24 h at room temperature. Co-evaporation with toluene afforded the corresponding acetyl derivative **7Ac** quantitatively as a pure oil. ^{1}H-NMR (250 MHz, $CDCl_3$) δ (ppm) = 7.34-7.27 (m, 10H), 5.50 (d, 1H, J = 4.9 Hz), 5.03 (t, 1H, J = 9.7 Hz), 4.89-4.54 (m, 5H), 4.28 (dd, 1H, J = 5.0, 2.4 Hz), 4.18 (dd, 1H, J = 8.0, 1.7 Hz) 3.88-3.74 (m, 2H), 3.52-3.42 (m, 2H), 3.41-3.29 (m, 1H), 3.15-3.06 (m, 1H), 2.08-2.01 (m, 1H), 2.00 (s, 3H), 1.65-1.53 (m, 1H), 1.52 (s, 3H), 1.43 (s, 3H), 1.32 (s, 6H), 1.15 (d, 1H, J = 12.0Hz), 1.06 (d, 3H, J = 6.5 Hz). ^{13}C-NMR (62.5 MHz, $CDCl_3$) δ (ppm) = 170.56, 138.65, 138.56, 128.55, 128.52, 128.23, 127.89, 127.71, 109.30, 108.69, 96.44, 86.14, 84.04, 78.79, 75.65, 75.47, 71.22, 70.73, 68.75, 67.22, 34.81, 33.41, 26.19, 26.10, 25.14, 24.48, 22.32, 21.25, 20.73, 18.08, 1.15. Anal. Calcd for $C_{35}H_{46}O_{10}$: C, 67.07; H, 7.40. Found: C, 67.12; H, 7.35. HPLC-MS analysis: retention time 11.03 min, ESI-MS (m/z), $[M+H]^+$ = 627.4, $[M+NH_4]^+$ = 644.4.

1,2;3,4-*O*-Diisopropylidene-6-*O*-(2-acetyl-3,4-di-*O*-benzyl-6-deoxy-5a-carba-α-D-2-mannosyl)-α-D-galactopyranoside (8Ac). To solution of pseudodisaccharide **8** in anhydrous pyridine (0.1 mL) were added at 0 °C 0.05 ml of acetic anhydride and the reaction was stirred for 24 h at room temperature. Co-evaporation with toluene afforded the corresponding acetyl derivative **8Ac** quantitatively as a pure oil. ^{1}H-NMR (250 MHz, $CDCl_3$) δ (ppm) = 7.36-7.26 (m, 10H), 5.52 (d, 1H, *J* =5.1 Hz), 5.12-5.05 (m, 1H), 4.91 (d, 1H, *J* = 10.6 Hz), 4.76-4.65 (m, 2H), 4.62-4.53 (m, 2H), 4.33-4.26 (m, 2H), 4.05-3.96 (m, 1H), 3.89-3.61 (m, 4H), 3.40 (t, 1H, *J* = 9.4 Hz), 1.96 (s, 3H), 1.87-1.68 (m,1H), 1.68-1.59 (m, 2H), 1.53 (s, 3H), 1.44 (s, 3H), 1.33 (s, 6H), 1.03 (d, 3H, *J* = 6.3 Hz). ^{13}C-NMR (62.5 MHz, $CDCl_3$) δ (ppm) = 175.84, 170.05, 166.54, 148.89, 139.08, 138.71, 137.00, 129.17, 128.46, 128.36, 128.26, 128.06, 127.72, 127.66, 125.43, 109.16, 108.59, 96.35, 82.96, 81.37, 75.53, 72.84, 70.85, 70.68, 70.64, 69.91, 69.44, 66.16, 32.43, 32.08, 29.82, 26.22, 26.09, 25.01, 24.45, 22.30, 21.28, 21.00, 18.18, 1.14. Anal. Calcd for $C_{35}H_{46}O_{10}$: C, 67.07; H, 7.40. Found: C, 67.15; H, 7.38. HPLC-MS analysis: retention time 11.37 min, ESI-MS (m/z), $[M+H]^+$ = 627.4, $[M+NH_4]^+$ = 644.4.

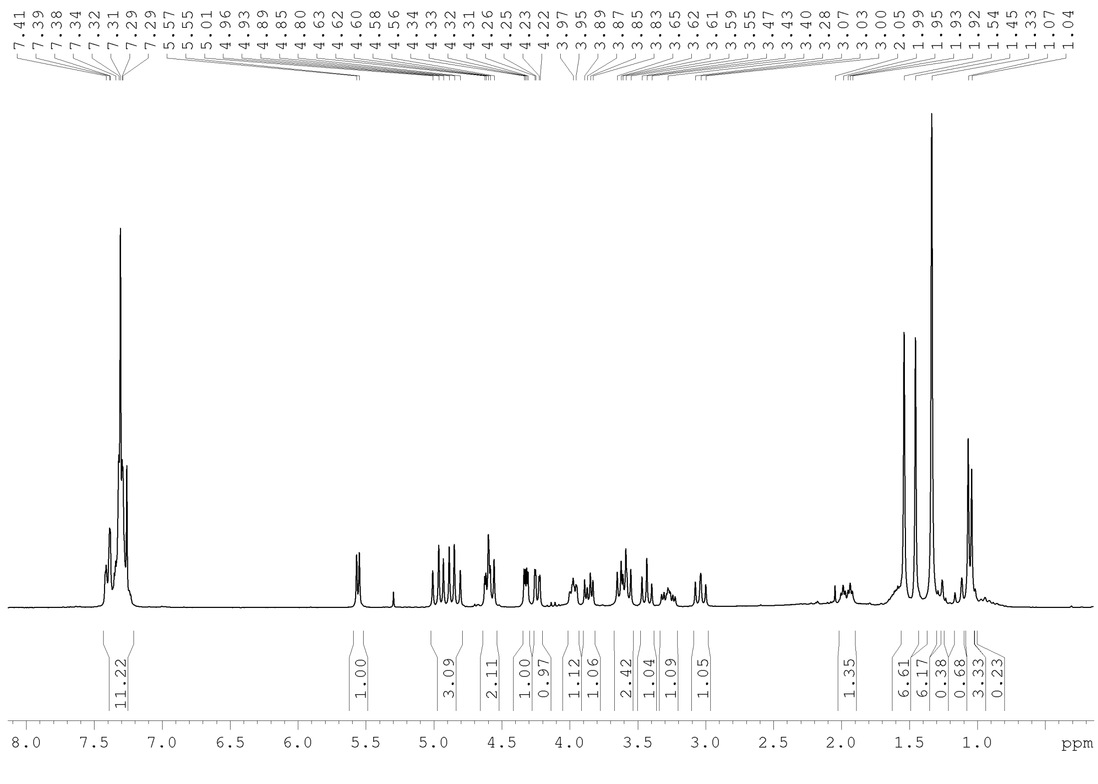

Figure S 14. ^{1}H-NMR spectrum of compound 7.

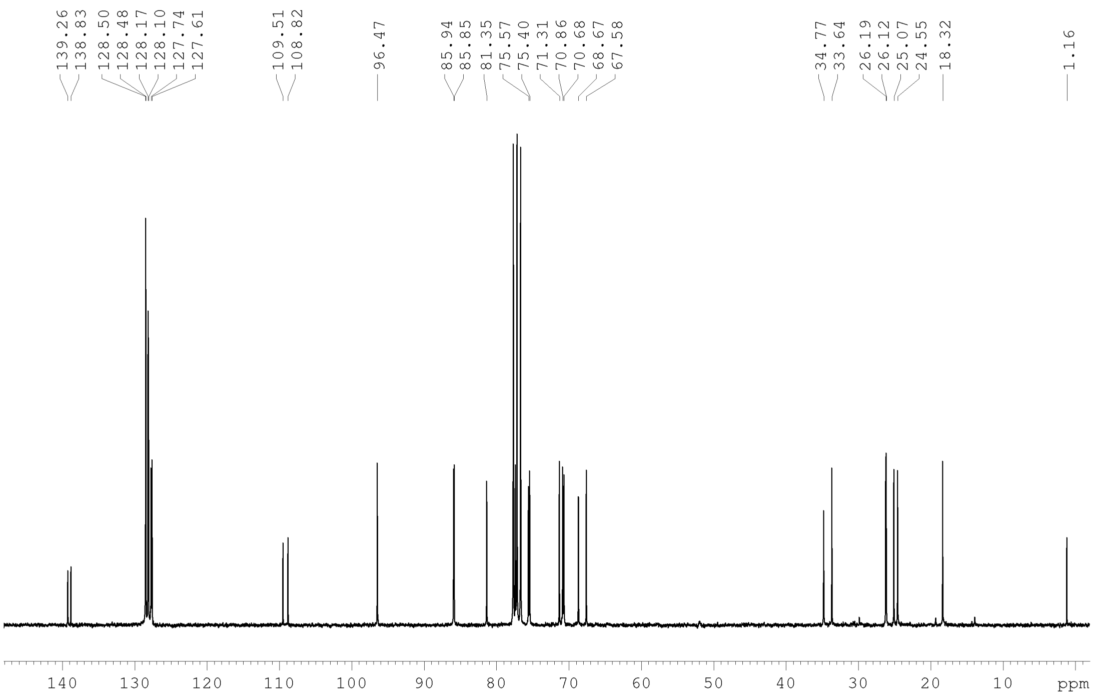

Figure S 15. ^{13}C-NMR spectrum of compound 7.

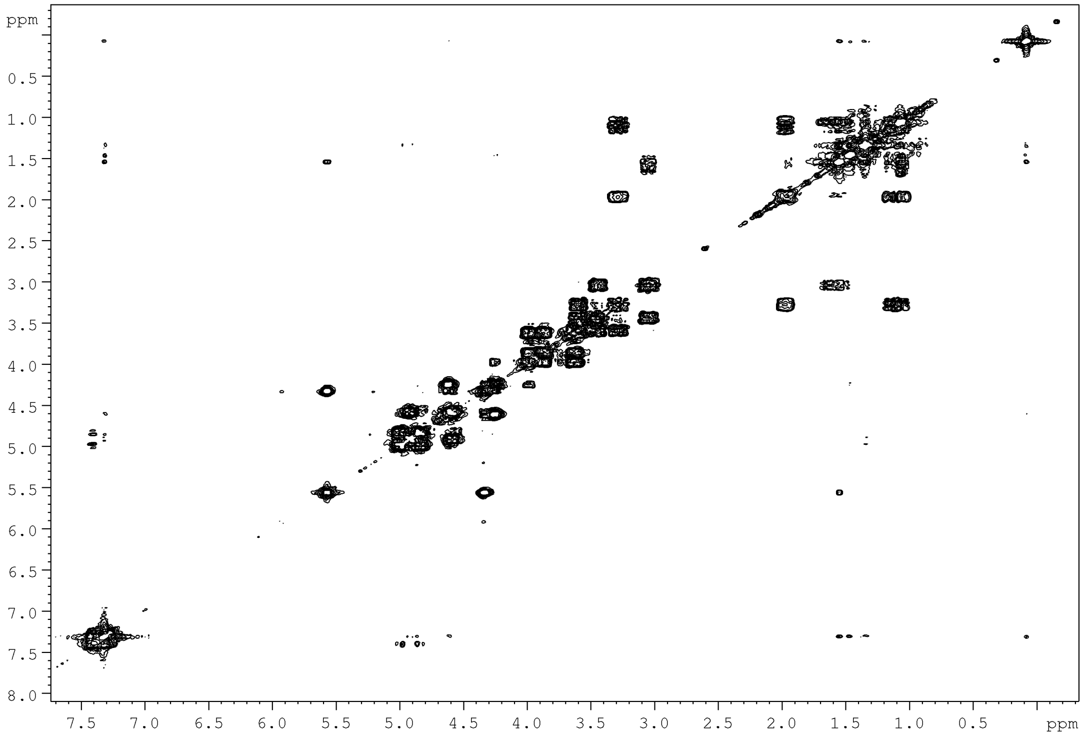

Figure S 16. COSY spectrum of compound 7.

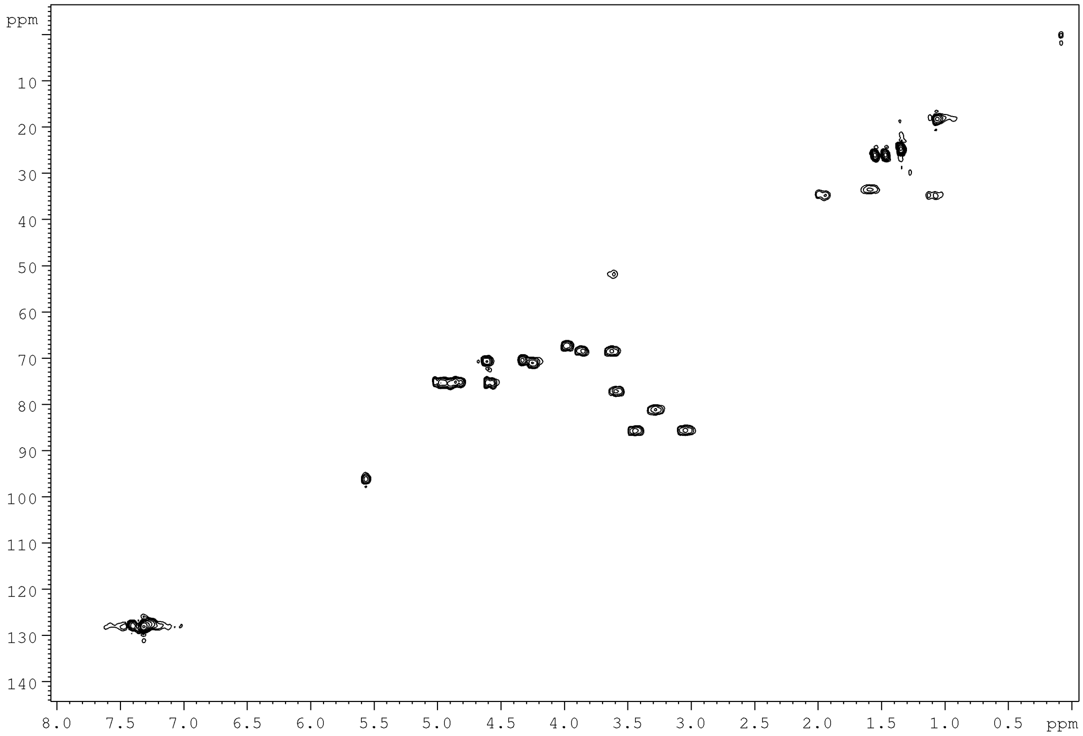

Figure S 17. HSQC spectrum of compound 7.

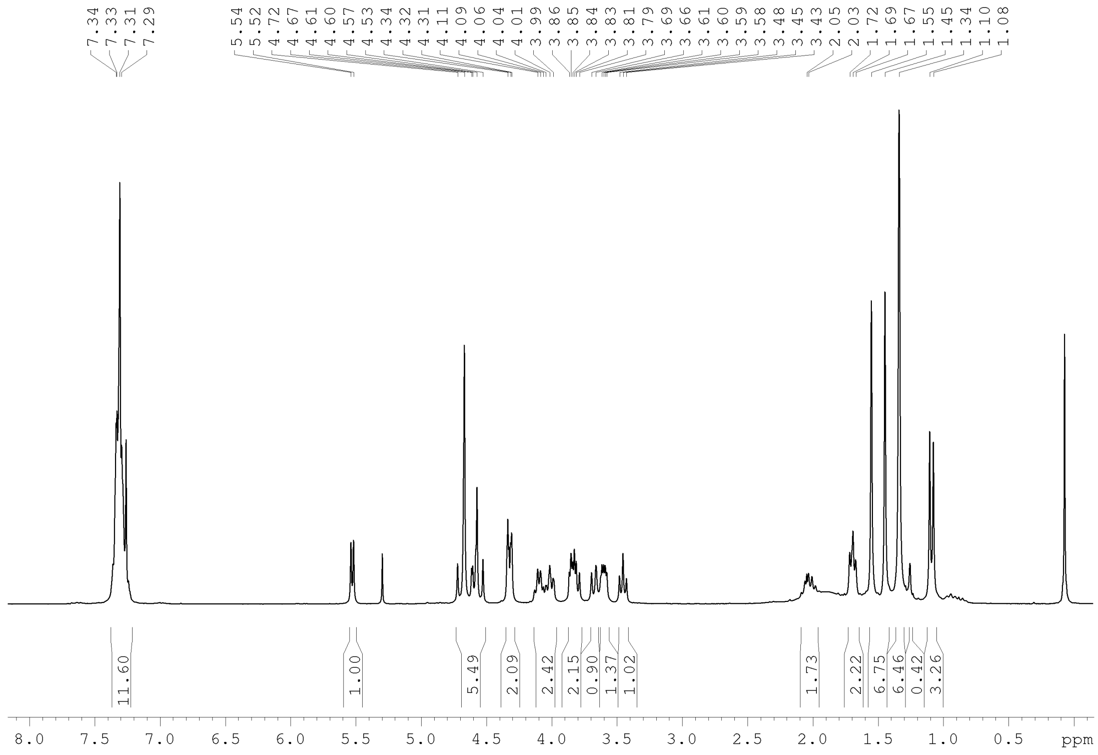

Figure S 18. ^{1}H-NMR spectrum of compound 8.

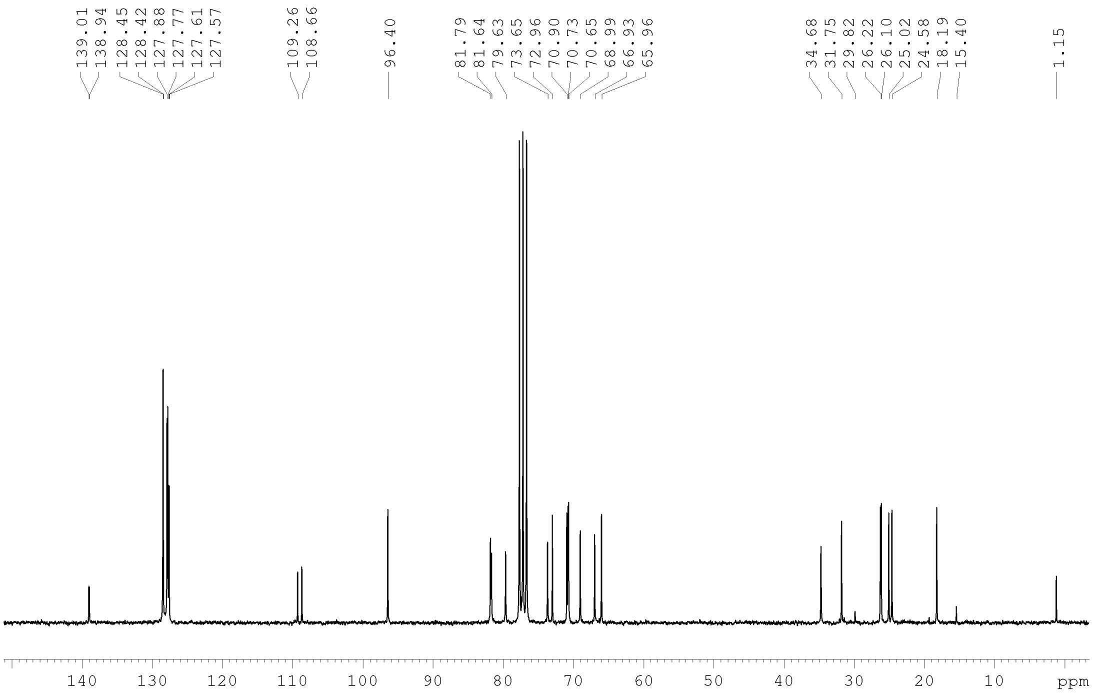

Figure S 19. ^{13}C-NMR spectrum of compound 8.

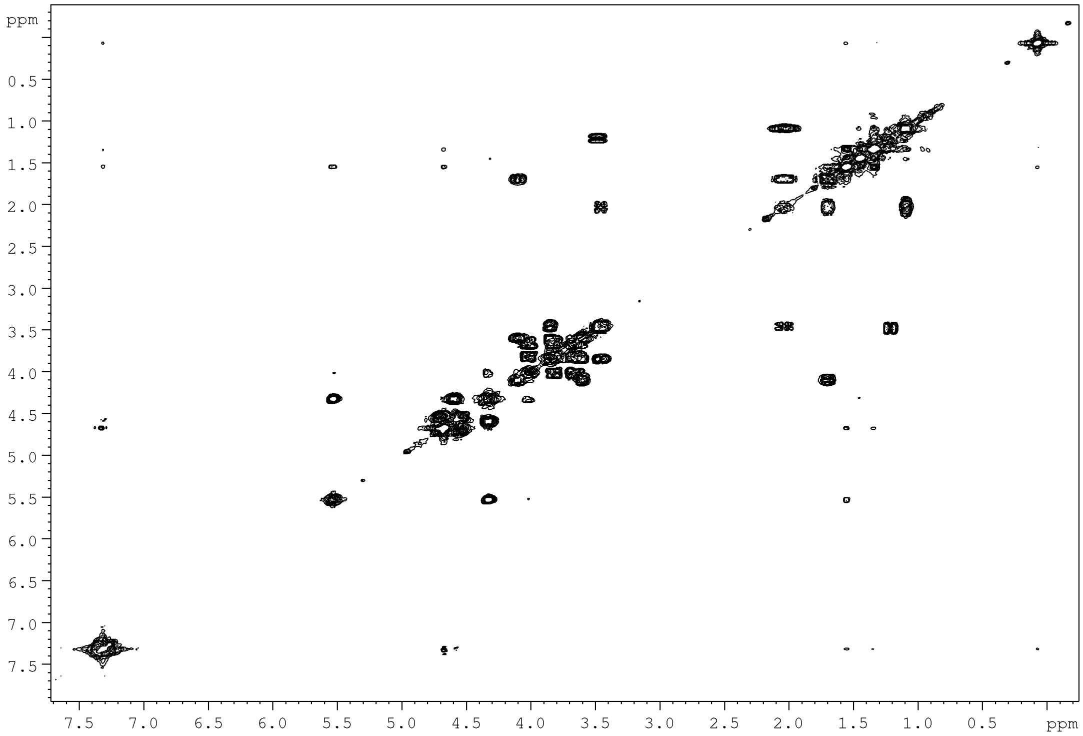

Figure S 20. COSY spectrum of compound 8.

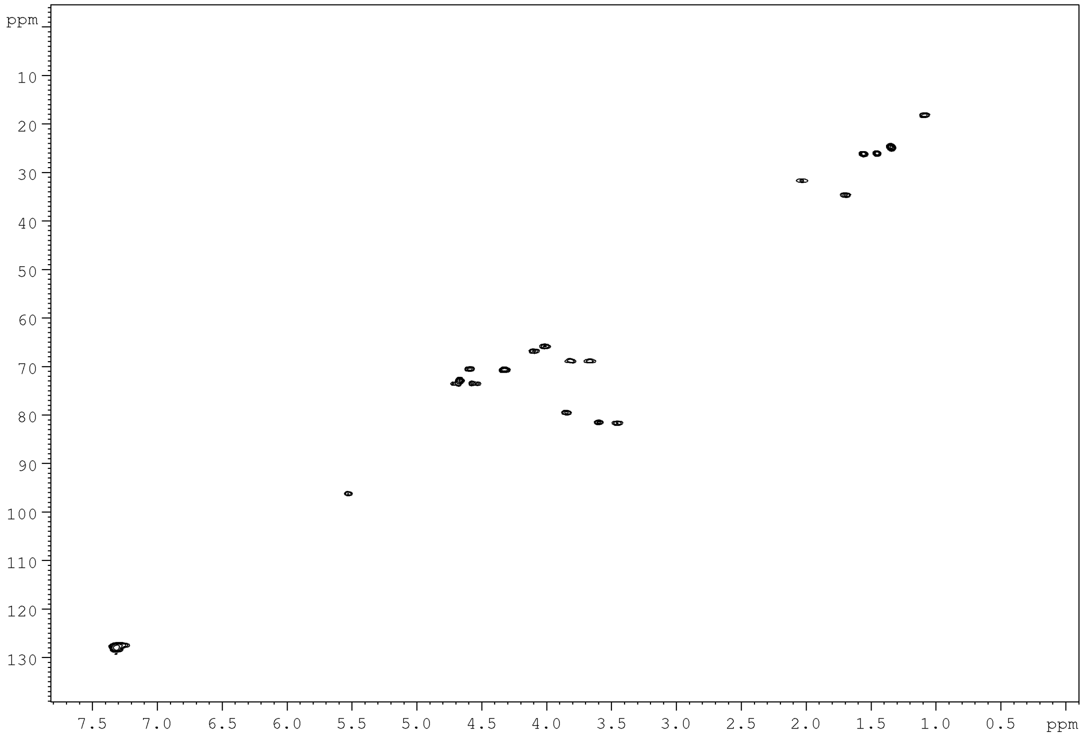

Figure S 21. HSQC spectrum of compound 8.

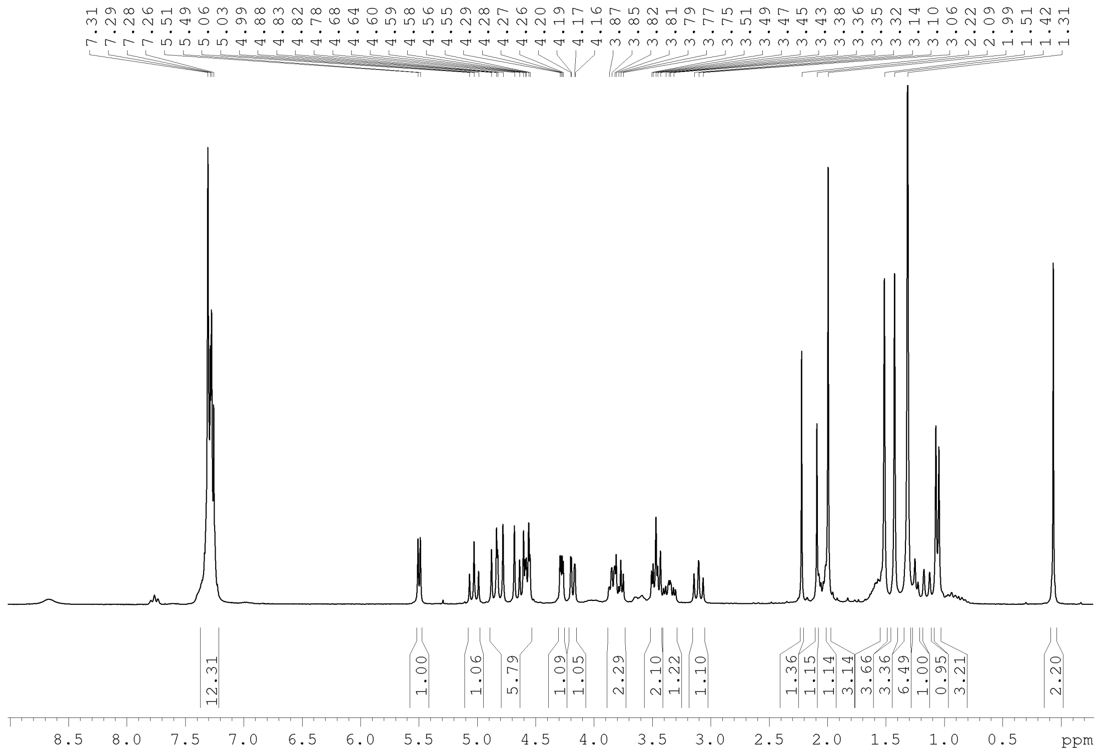

Figure S 22. ^{1}H-NMR spectrum of compound 7Ac.

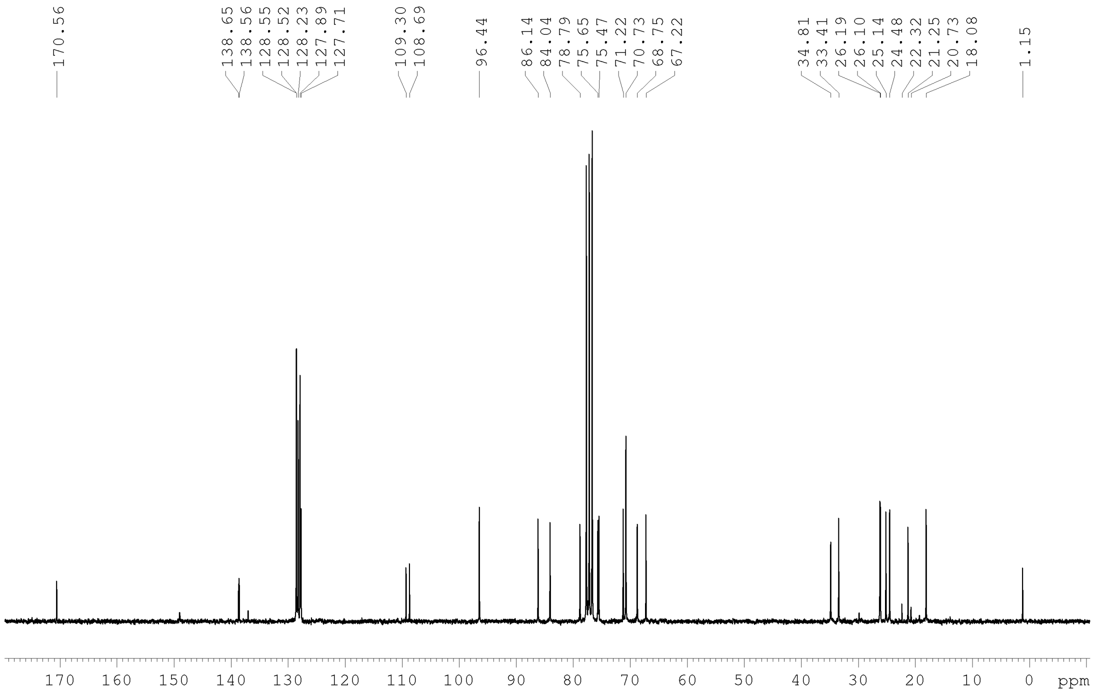

Figure S 23. ^{13}C-NMR spectrum of compound 7Ac.

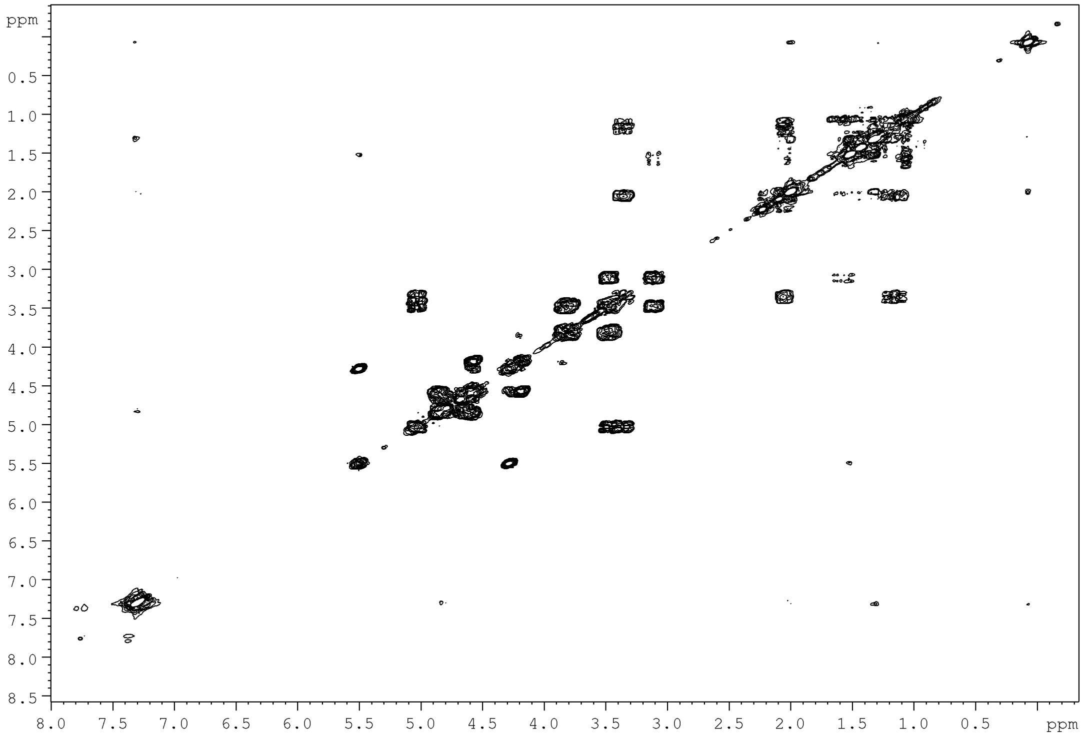

Figure S 24. COSY spectrum of compound 7Ac.

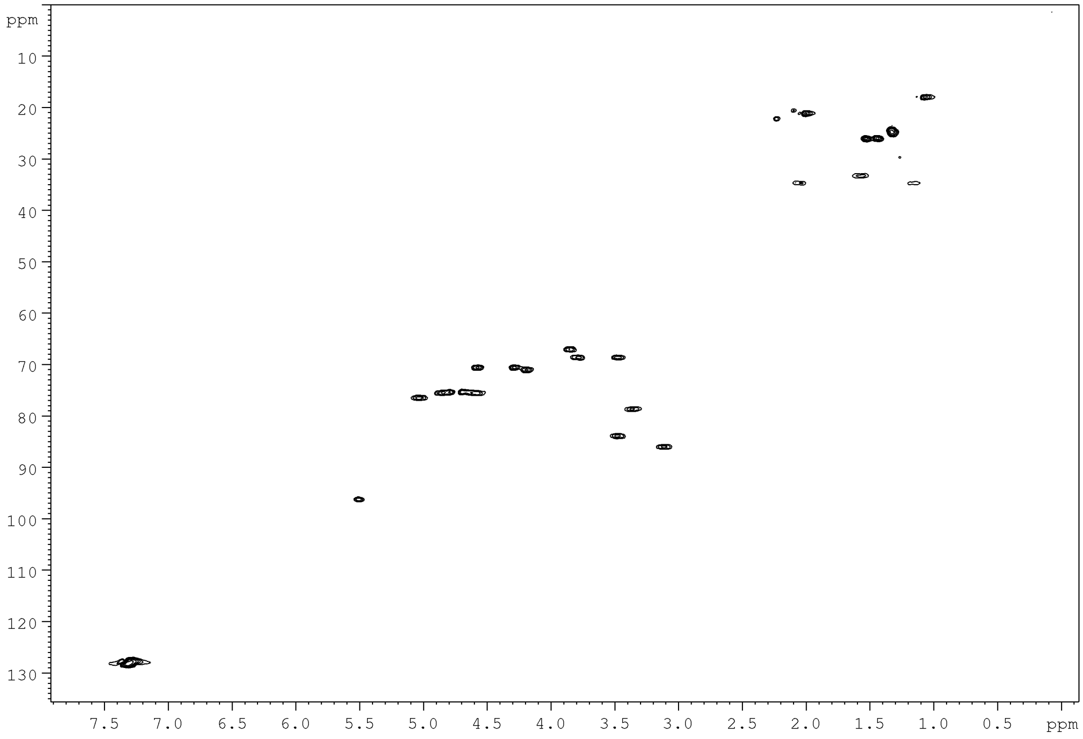

Figure S 25. HSQC spectrum of compound 7Ac.

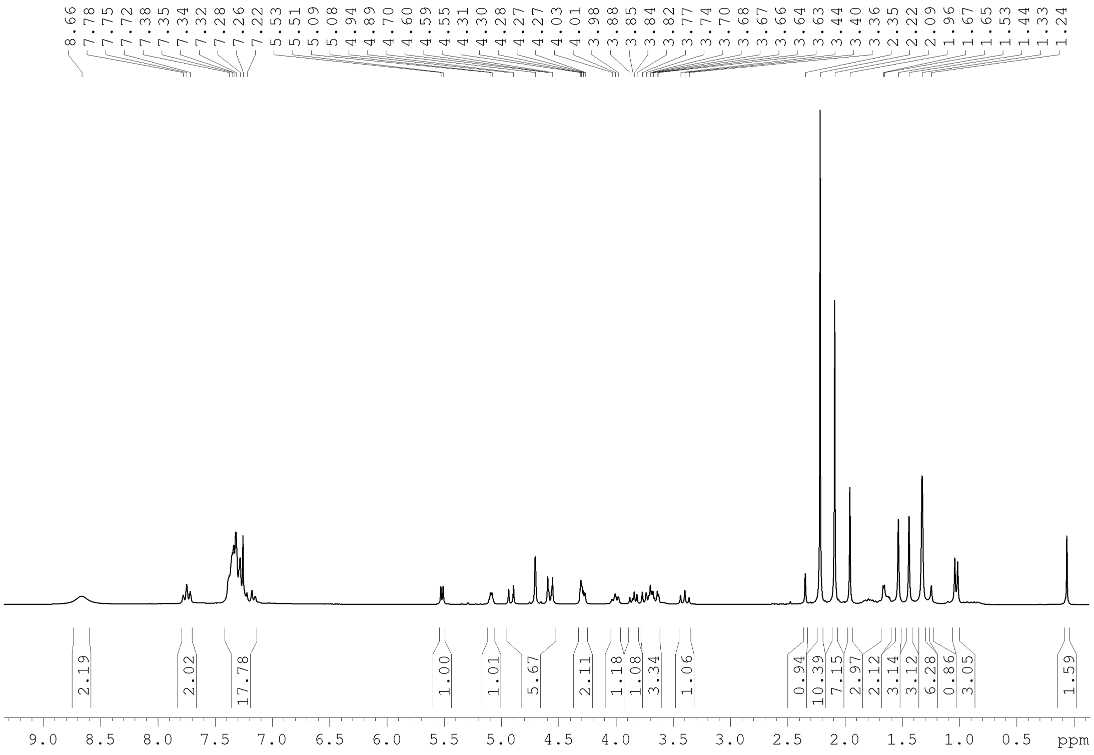

Figure S 26. ^{1}H-NMR spectrum of compound 8Ac.

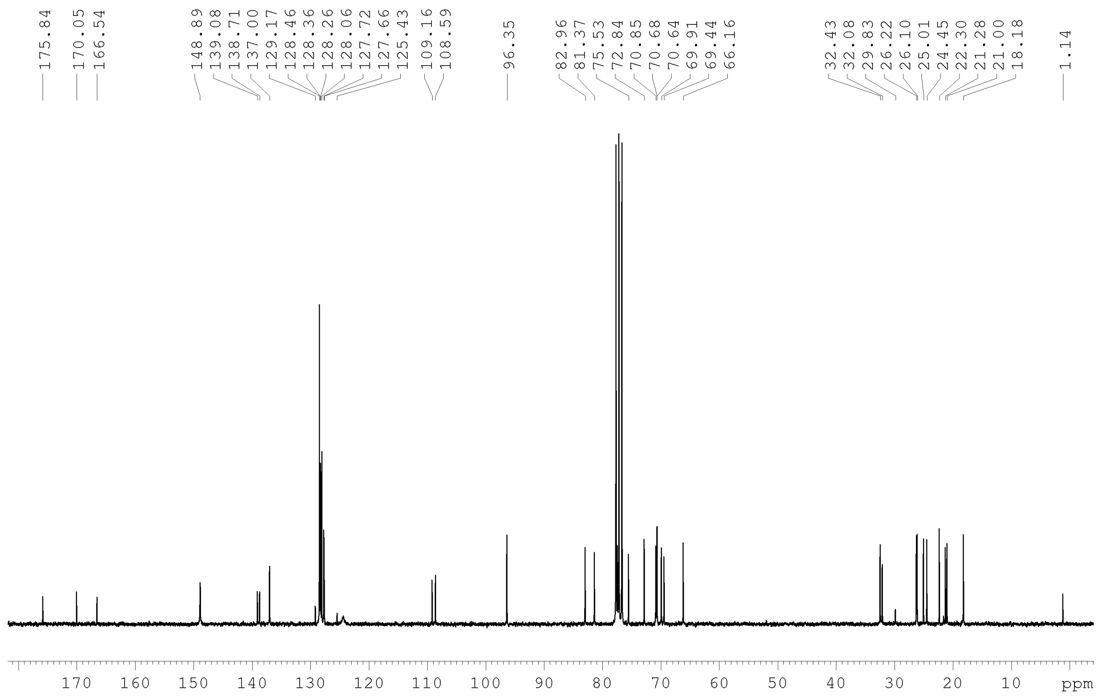

Figure S 27. ^{13}C-NMR spectrum of compound 8Ac.

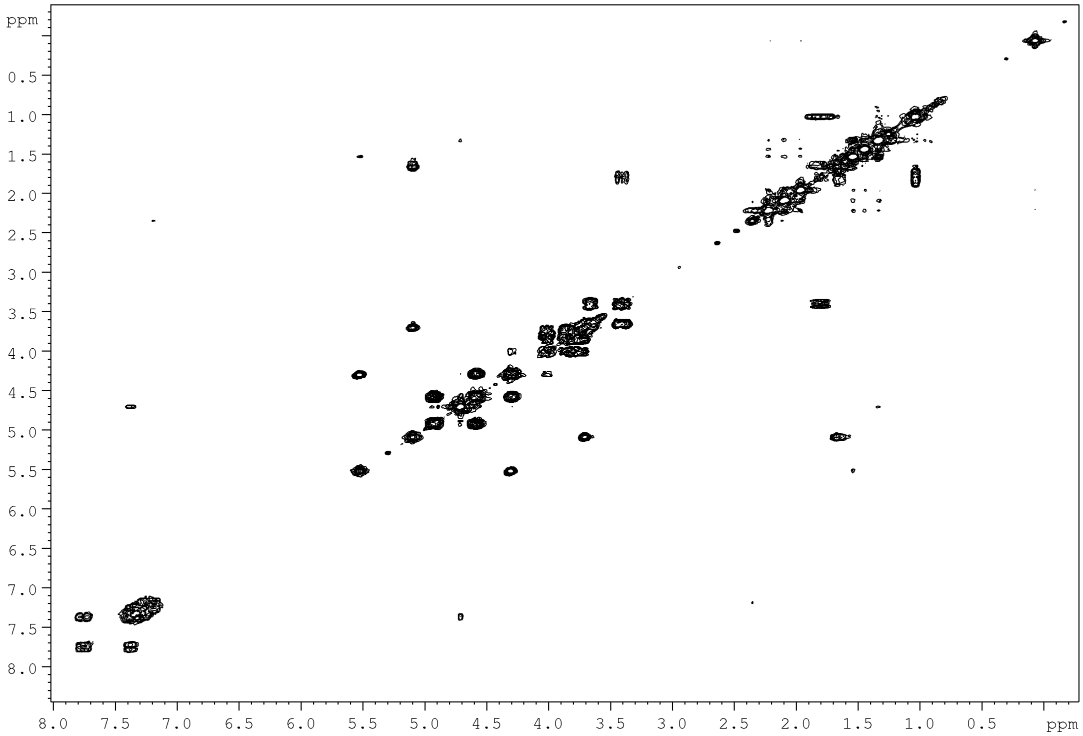

Figure S 28. COSY spectrum of compound 8Ac.

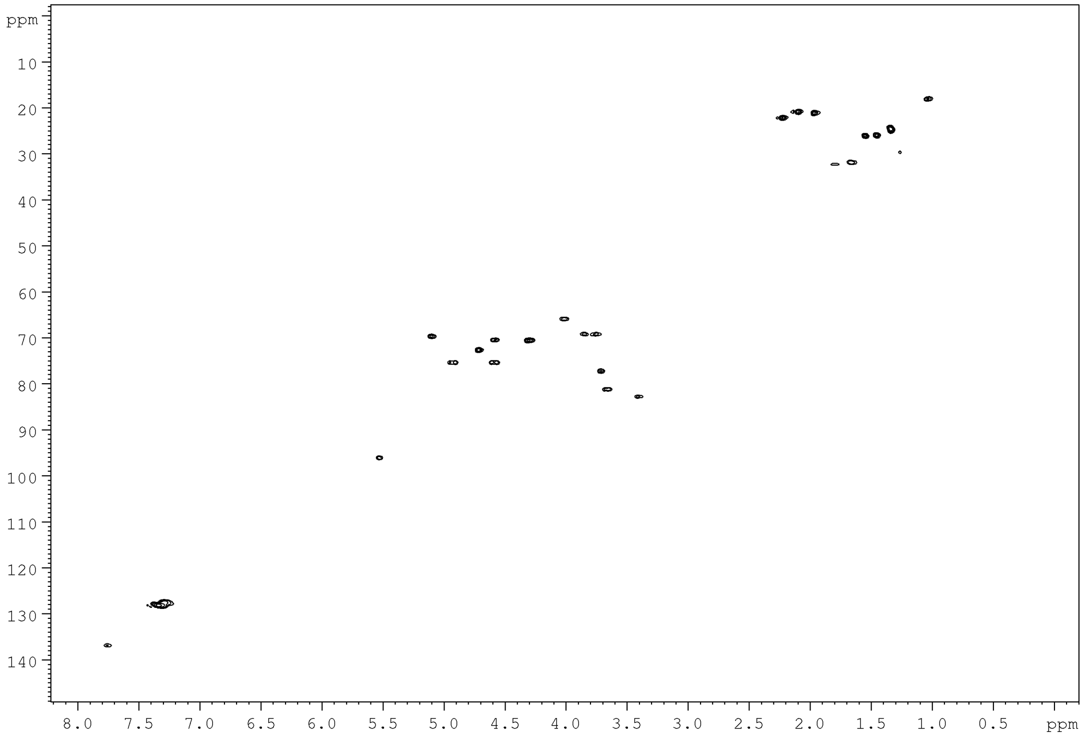

Figure S 29. HSQC spectrum of compound 8Ac.

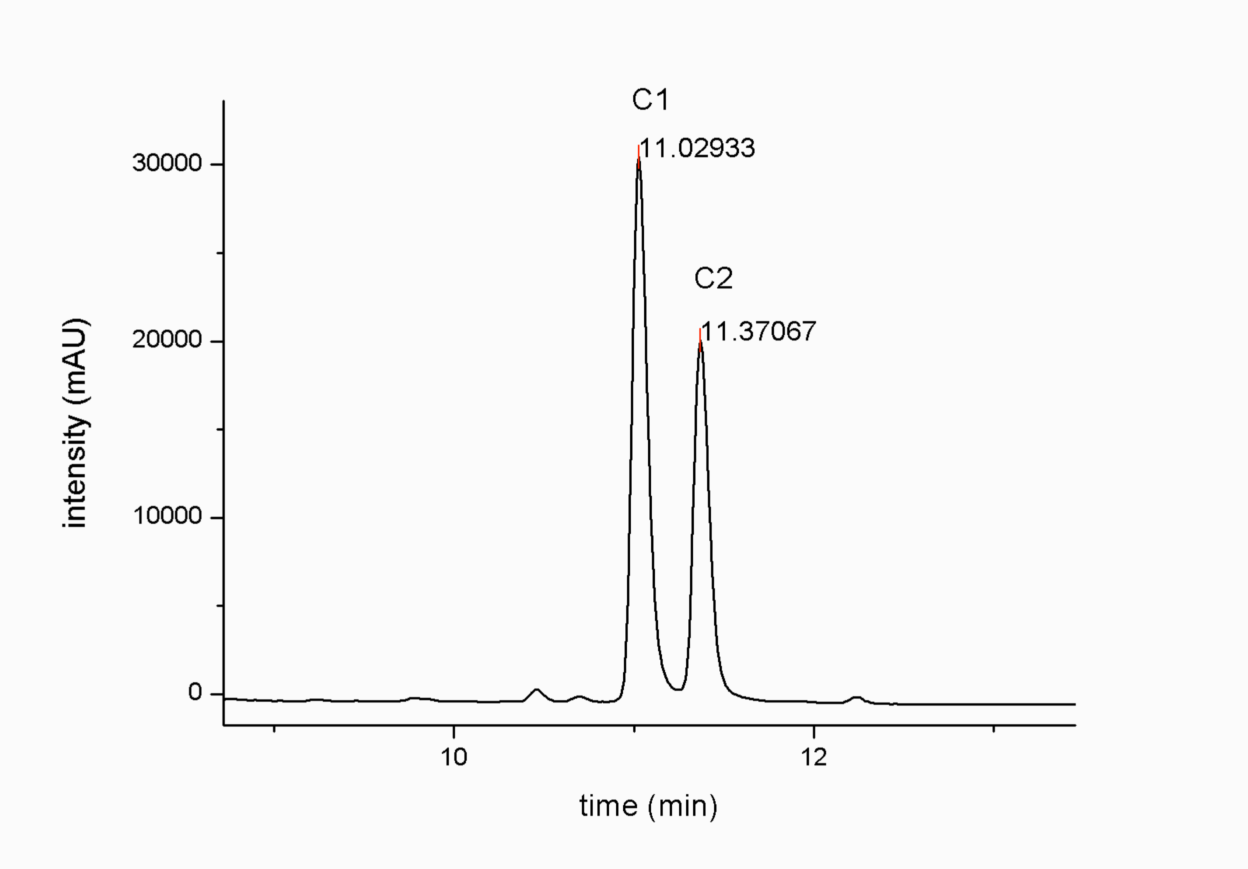

Figure S 30. HPLC chromatogram of a mixture of standard 7Ac and 8Ac.

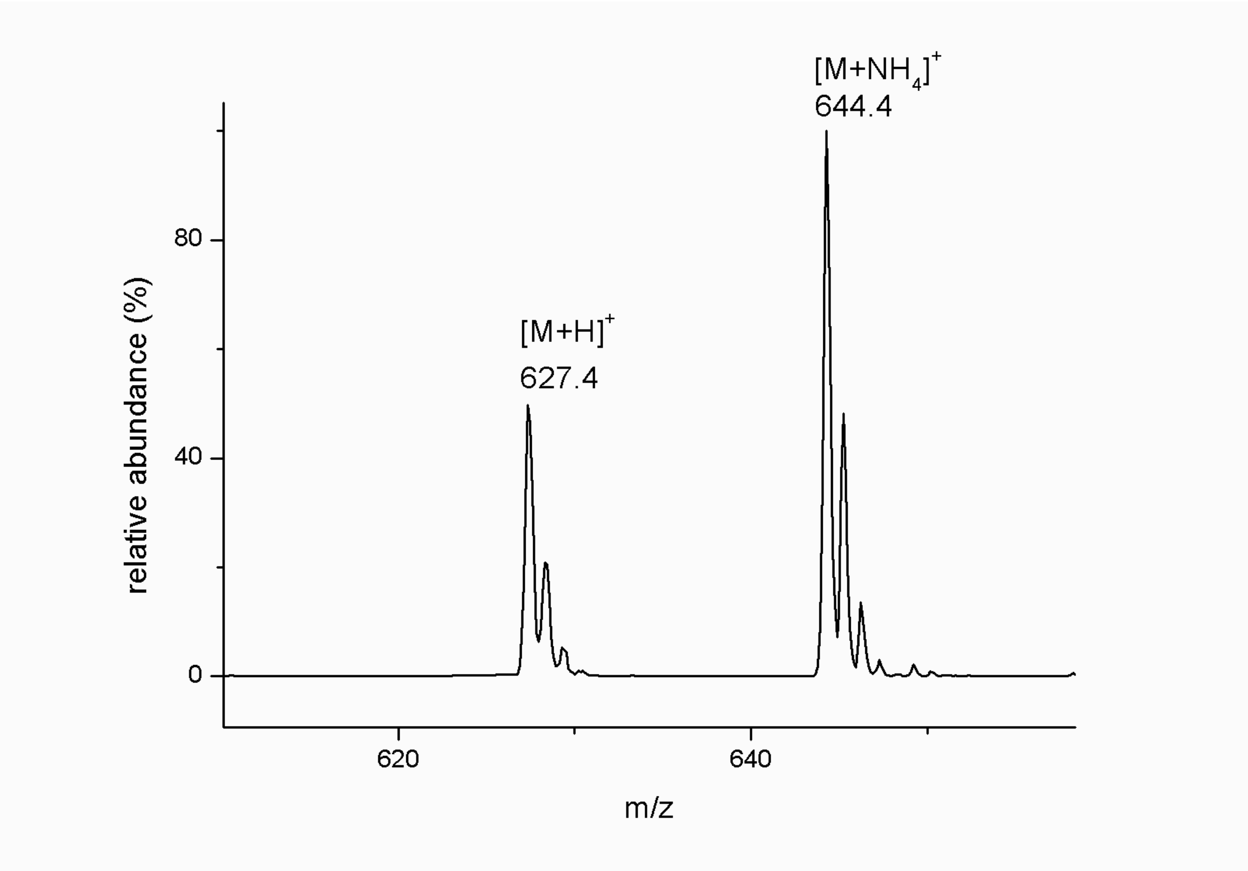

Figure S 31. ESI-MS spectrum of the mixture of 7Ac and 8Ac.

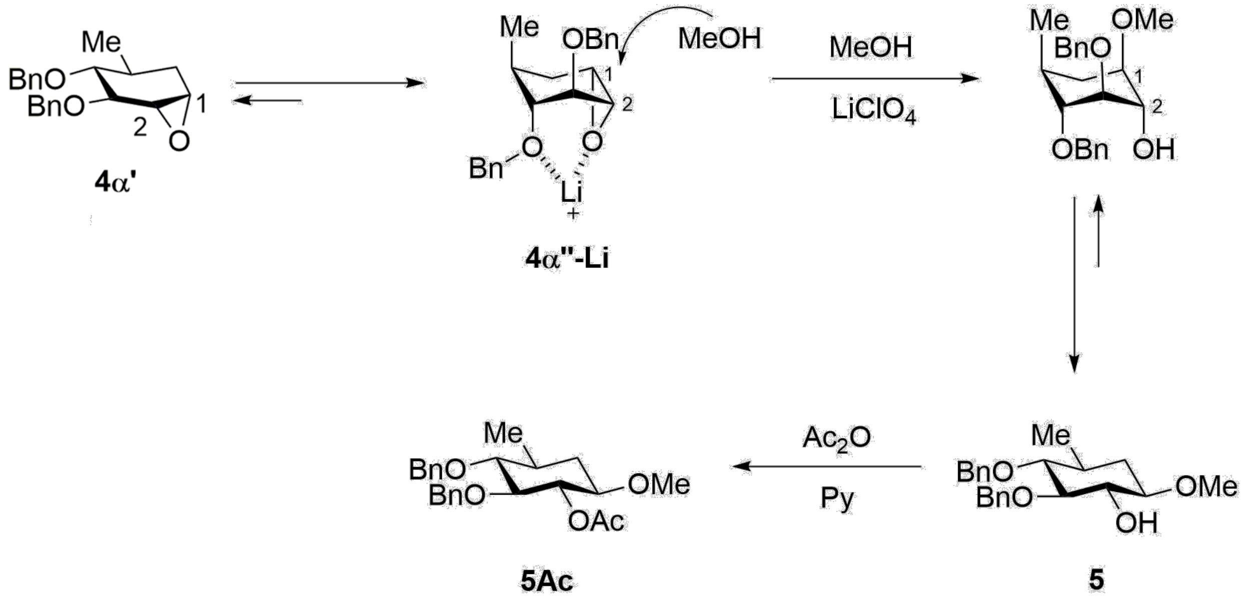

Scheme S3. $LiClO_4$ catalyzed reaction on 4α.

Reaction of epoxide 4α under $LiClO_4$/MeOH protocol. A solution of epoxide **4α** (0.012 g, 0.037 mmol, 1.0 equiv-) in methanol (1.0 mL) was added to a 5.0 M solution of $LiClO_4$ in methanol and the resulting reaction mixture was stirred for 7 days at 80 °C. After dilution with Et_2O, the organic phase was washed with water and dried with anhydrous magnesium sulfate and the solvent evaporated. The crude product consisted exclusively of the alcohol **5** (^{1}H NMR). The crude was purified by preparative TLC (SiO_2, hexane : ethyl acetate = 7:3) to afford the methoxy alcohol **5** as a pure colorless liquid (0.007 g, yield = 55%). **2-Hydroxy-6-deoxy-3,4-di-*O*-benzyl-1-*O*-methyl-5a-carba-α-D-glucopyranoside (5).** R_f = 0.24 (hexane : ethyl acetate = 7:3). ^{1}H-NMR (250 MHz, $CDCl_3$) δ (ppm) = 7.42-7.26 (m, 10H), 4.91 (d, 1H, *J* = 10.8 Hz), 4.91 (d, 1H, *J* = 11.2 Hz), 4.86 (d, 1H, *J* = 11.2 Hz), 4.61 (d, 1H, *J* = 10.8 Hz), 3.57-3.34 (m, 2H), 3.43 (s, 3H), 3.17-3.01 (m, 2H), 2.04 (dt, 1H, *J* = 13.2, 4.0 Hz), 1.71-1.51 (m, 2H), 1.08 (d, 3H, *J* = 6.5 Hz). ^{13}C-NMR (62.5 MHz, $CDCl_3$) δ (ppm) = 139.0, 138.7, 128.7,128.6, 128.5, 128.2, 128.1, 127.9, 86.1, 85.9, 81.0, 76.7, 75.6, 75.5, 57.4, 34.0, 33.7, 18.3.

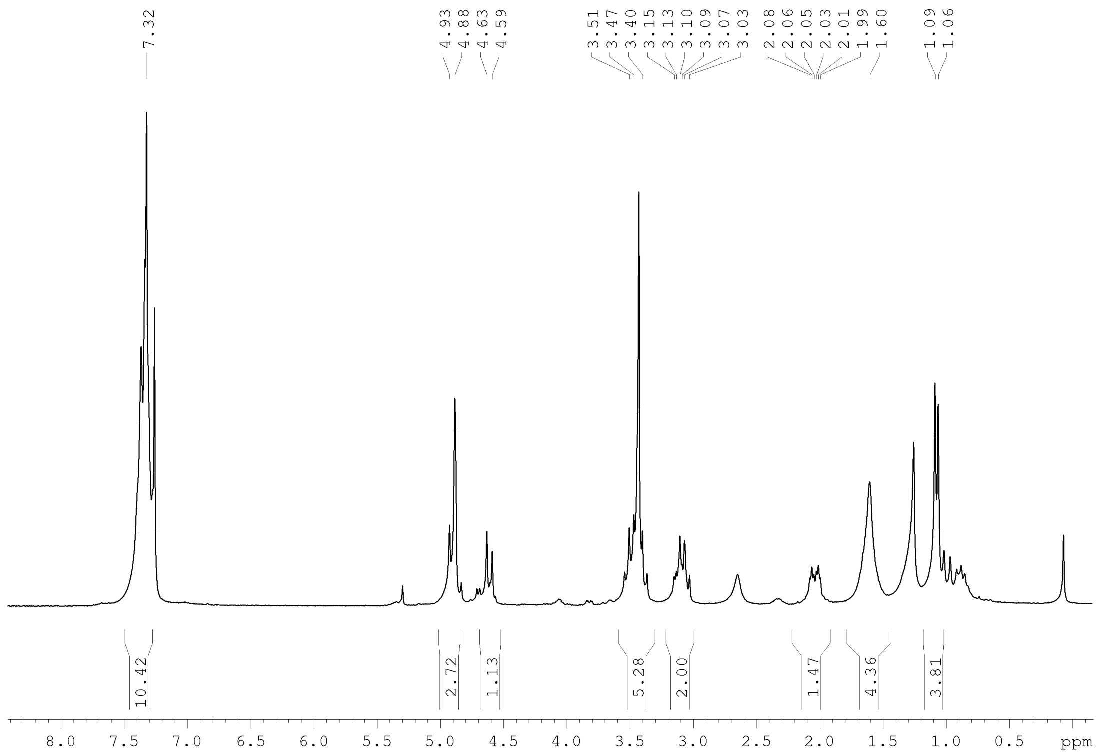

Figure S 32. ^{1}H-NMR spectrum of compound 5.

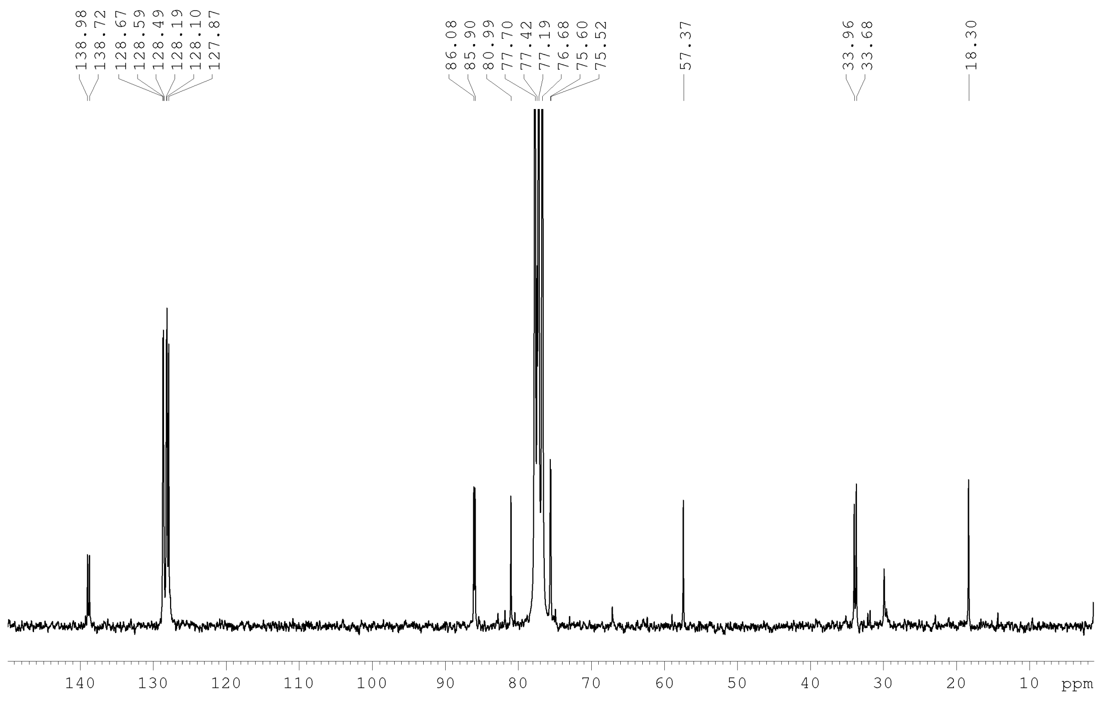

Figure S 33. ^{13}C-NMR spectrum of compound 5.

Reaction of epoxide 4α with MeOH/[Li(G3)]TFSI (entry 1 Table 2). Epoxide **4α** (20.0 mg, 0.062 mmol, 1 eq) was added to a solution of **[Li(G3)]TFSI** (1.2 mL), which was previously dried under vacuum at 70 °C for 24 h. Subsequently MeOH (0.05 mL, 1.24 mmol, 20.0 eq) was added to the reaction mixture, which was stirred at 70 °C for 3 days. Only the starting material was recovered (^{1}H-NMR).

Reaction of epoxide 4α with MeOH/[Li(G4)]TFSI (entry 2 Table 2). Epoxide **4α** (20.0 mg, 0.062 mmol, 1 eq) was added to a solution of **[Li(G4)]TFSI** (1.2 mL), which was previously dried under vacuum at 70 °C for 24 h. Subsequently MeOH (0.0125 mL, 0.31 mmol, 5.0 eq) was added to the reaction mixture, which was stirred at 70 °C for 24 h. After dilution with ethyl acetate, the mixture was washed with brine (twice) and the organic layer was dried with anhydrous magnesium sulfate and the solvent evaporated. The crude product consisted in a 60:40 mixture of the two regioisomeric *trans*-methoxy alcohols **5** and **6** (^{1}H-NMR).

Reaction of epoxide 4α with MeOH/[Li(G4)]TFSI /excess of $LiTf_2N$ (entry 3 Table 2). An excess of $LiTf_2N$ (5% m/m) was added to 1 ml of IL **[Li(G4)]TFSI** and the solution was dried

under vacuum at 70 °C. Epoxide **4α** (22.0 mg, 0.068, 1 eq) was added to the IL and subsequently MeOH (0.014 mL, 0.34 mmol, 5.0 eq) was finally added to the reaction mixture, which was stirred at 70 °C for 3 days. After dilution with diethyl ether and filtration on a silica gel pad, the solvent was evaporated to afford a crude product (12 mg) consisting in a 60:40 mixture of the two regioisomeric *trans*-methoxy alcohols **5** and **6** (^{1}H-NMR).

Reaction of epoxide 4α with diaceton-D-galactose/[Li(G4)]TFSI (entry 4 Table 2). Epoxide **4α** (31 mg, 0.096 mmol, 1 eq) was added to a solution of **[Li(G4)]TFSI** (2 mL), which was previously dried under vacuum at 70 °C for 24 h. Subsequently, **diaceton-D-galactose** (0.288 mmol, 3 eq) was added to the reaction mixture, which was stirred at 70 °C for 3 days. Then, the reaction was cooled down and a saturated solution of lithium chloride was added, which was extracted with chloroform. The organic phase was washed with saturated lithium chloride, it was dried with anhydrous sodium sulfate and the solvent was evaporated. The crude product (70 mg) was dissolved in 5 ml of pyridine and 2 ml of freshly distilled acetic anhydride and stirred overnight. The solvents were co-evaporated with toluene to afford a crude, which was subjected to HPLC-MS analysis. The sample consisted of a 80:20 regioisomeric mixture of respectively **7Ac** (C1 adduct) and **8Ac** (C2 adduct) and the peaks were attributed using as standards the same compounds obtained and characterized in the $Cu(OTf)_2/CH_2Cl_2$ process. ESI-MS of each peak (m/z), $[M+H]^+ = 627.4$, $[M+NH_4]^+ = 644.4$.

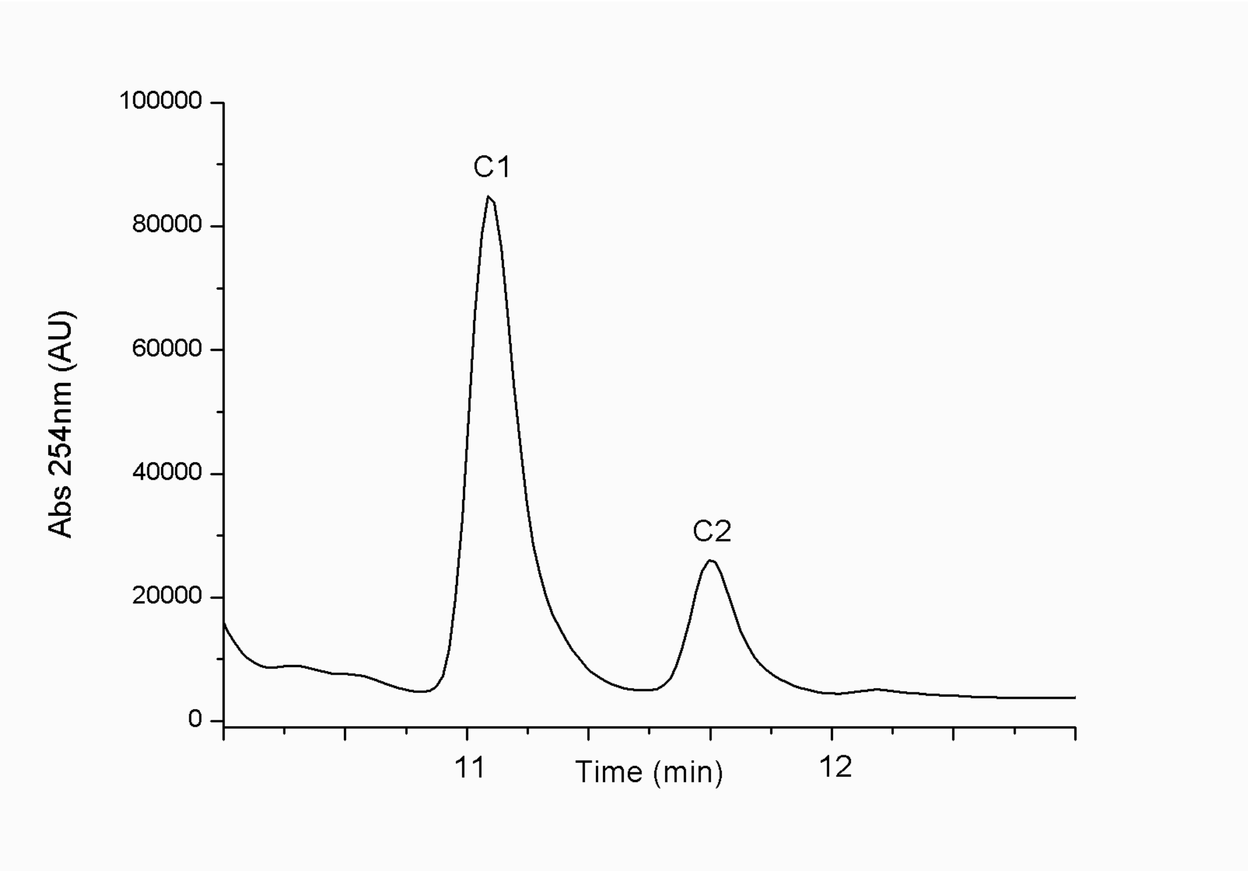

Figure S 34. HPLC chromatogram of the acetylated crude of the reaction of 4α with diaceton-D-galactose/ [Li(G4)]TFSI.
